# Supplementary material for: Systemic Metabolomic Profiles in Adult Patients with Bacterial Sepsis: Characterization of Patient Heterogeneity at the Time of Diagnosis
Source: Biomolecules. 2023 Jan 24;13(2):223. doi: 10.3390/biom13020223 (PMC9953377; doi:10.3390/biom13020223)
Supplement: Supplementary file 1 [file biomolecules-13-00223-s001.zip › biomolecules-2131124-supplementary.pdf]

Supplementary information

Systemic metabolomic profiles in adult patients with bacterial sepsis; characterization of patient heterogeneity at the time of diagnosis

Knut Anders Mosevoll, Bent Are Hansen, Ingunn Margareetta Gundersen, Håkon Reikvam, Øyvind Bruserud, Øystein Bruserud and Øystein Wendelbo

Table S1: Clinical characteristics of the patients included in our present study; a comparison of the Sepsis-3 (35 patients) patients and patients that only fulfilled the Sepsis-2 criteria (25 patients); Table S2: Classification of individual metabolites detected in our present study; Table S3: Metabolic differences of individual metabolites between patients fulfilling the Sepsis-3 definition (PMID 26903338) versus patients only fulfilling the Sepsis-2 criteria (PMID 12682500, 12664219); Table S4: Metabolic differences of individual metabolites between patients with and without bacteremia; Table S5: Differences of individual metabolites between patients Gram negative versus Gram positive infection; Table S6: Summary of individual metabolites between patients with Gram negative and Gram positive infection; Table S7: Subclassification of the 42 metabolites showing the strongest correlation with total SOFA score, i.e. a p-value <0.05 and a correlation factor >0.6 when using the Pearson test; Table S8: The correlation between the serum level of individual metabolites and the total SOFA score; a list presenting all metabolites that showed a p-value corresponding to <0.05 when using the Pearson's correlation test; Table S9: Correlation between individual the 56 steroid hormone metabolites and the maximal total SOFA score; Figure S1: Subclassification of sepsis patients based on lipid metabolites that showed a strong correlation with the total SOFA score; Methodological strategies used by Metabolon for metabolomic nalyses of our present samples.

Table S1. Clinical characteristics of the patients included in our present study; a comparison of the Sepsis-3 (35 patients) patients and patients that only fulfilled the Sepsis-2 criteria (25 patients). Statistically significant differences are marked with grey and p-values in bold.

	Sepsis 3.0 (n=35)		*Sepsis 2.0 (n=25)*		*p-value*
	Median	*Variation range*	*Median*	*Variation range*	
**Age (years)**[a]	66	23-96	62	20-84	0.412
**Peripheral blood examination**[a]					
Hgb (g/dL)	13	8.7-16.2	12.8	10.7-17.5	0.929
WBC (10^9/L)	12.5	4.9-46.6	15.9	8.4-32.6	**0.030**
Neutrophils (10^9/L)	10.8	3.4-41.1	13.6	5.2-28.6	0.196
CRP (mg/L)	169	4-538	182	61-370	0.313
Erythrocyte sedimentation rate (ESR)	36	5-126	47	17-107	0.124
Creatinine (umol/L)	114	51-475	69	27-706	**0.001**
**Clinical findings at hospitalization**[a]					
Temperature (°C)	38.8	36.3-41.4	38.4	36.5-41.5	0.305
Heart rate (/min)	111	74-140	108	70-131	0.319
Respiratory frequency (/min)	24	14-60	24	15-53	0.906
Systolic blood pressure (mmHg)	121	11-161	131	104-180	0.131
Diastolic blood pressure (mmHg)	66	26-110	78	41-97	0.410
Glascow coma scale (GCS)	15	3-15	15	15-15	0.062
**Observations during hospitalization**					
Lowest systolic blood pressure (mmHg)	88	65-124	109	91-134	**<0.001**
Lowest middle artery pressure (mmHg)	59	50-96	68	61-105	**0.015**
PaO2 (kPa)	8.4	4.6-17.9	9.4	5.6-15.2	0.198
FiO2	0.21	0.21-0.42	0.21	0.21-0.21	0.592
PaO2/FiO2	38	11-85	45	26.6-72	0.067
Total SOFA≥2[b.c]	35	100%	0	0	**<0.0005**
Any organ failure SOFA≥2	28	80%	0	0	**<0.0005**
Respiratory failure	27	77%	8	32%	**0.0006**
Hypotension	24	69%	7	28%	**0.002**
Platelets <100 x 10^9/L	13	37%	0	0%	**0.001**
Renal failure	17	49%	0	0%	**<0.001**
Liver failure	9	26%	2	8%	0.08
CNS failure	8	23%	0	7%	**0.01**
**Infection site**[b]					0.075
Urinary tract	14	40%	14	56%	ns
Respiratory	4	11%	5	20%	ns
Soft tissue	8	23%	3	12%	ns
CNS	3	9%	0	0	ns
Endocarditis	5	14%	0	0	ns
Other	1	2%	3	12%	ns

[a]Median (min–max). Mann–Whitney U test; ns, not significant.
[b]N(%). χ^2 test; ns, not significant.
[c]Defined as increase in sequential (sepsis-related) organ failure assessment score ≥2, sepsis induced (pre-existing, stable organ failure not included).

Table S2. Classification of individual metabolites detected in our present study. The table present the main metabolic classes (capitals in bold), the various subclasses and for each subclass the number of idividual metabolites (number in parethesis).

**AMINO ACIDS METABOLISM**
Glycine, Serine and Threonine Metabolism (10)
Alanine and Aspartate Metabolism (9)
Glutamate Metabolism (12)
Histidine Metabolism (19)
Lysine Metabolism (19)
Phenylalanine Metabolism (7)
Tyrosine Metabolism (21)
Tryptophan Metabolism (25)
Leucine, Isoleucine and Valine Metabolism (33)
Methionine, Cysteine, SAM and Taurine Metabolism (26)
Urea cycle; Arginine and Proline Metabolism (24)
Creatine Metabolism (3)
Polyamine Metabolism (9)
Guanidino and Acetamido Metabolism (3)
Glutathione Metabolism (7)
**PEPTIDE**
Gamma-glutamyl Amino Acid (18)
Dipeptide (11)
Polypeptide (2)
Fibrinogen Cleavage Peptide (14)
Acetylated Peptides (4)
Modified Peptides (1)
**CARBOHYDRATE**
Glycolysis, Gluconeogenesis, and Pyruvate Metabolism (5)
Pentose Metabolism (8)
Glycogen Metabolism (1)
Disaccharides and Oligosaccharides (2)
Fructose, Mannose and Galactose Metabolism (4)
Aminosugar Metabolism (5)
Aminosugar Metabolism (4)
Advanced Glycation End-product (1)
**ENERGY METABOLISM**
TCA Cycle (9)
Oxidative Phosphorylation (1)
**LIPID METABOLISM**
Fatty Acid Synthesis (2)
Short Chain Fatty Acid (2)
Medium Chain Fatty Acid (10)
Long Chain Saturated Fatty Acid (7)
Long Chain Monounsaturated Fatty Acid (7)
Long Chain Polyunsaturated Fatty Acid (n3 and n6) (19)

Fatty Acid, Branched (7)
Fatty Acid, Dicarboxylate (33)
Fatty Acid, Amino (3)
Fatty Acid Metabolism (also BCAA Metabolism) (4)
Fatty Acid Metabolism (Acyl Glutamine) (2)
Fatty Acid Metabolism (Acyl Glycine) (8)
Fatty Acid Metabolism (Acyl Carnitine, Short Chain) (1)
Fatty Acid Metabolism (Acyl Carnitine, Medium Chain) (7)
Fatty Acid Metabolism (Acyl Carnitine, Long Chain Saturated) (7)
Fatty Acid Metabolism (Acyl Carnitine, Monounsaturated) (10)
Fatty Acid Metabolism (Acyl Carnitine, Polyunsaturated) (6)
Fatty Acid Metabolism (Acyl Carnitine, Dicarboxylate) (5)
Fatty Acid Metabolism (Acyl Carnitine, Hydroxy) (5)
Carnitine Metabolism (2)
Ketone Bodies (2)
Fatty Acid Metabolism (Acyl Choline) (9)
Fatty Acid, Monohydroxy (23)
Fatty Acid, Dihydroxy (5)
Docosanoid (1)
Eicosanoid (5)
Endocannabinoid (10)
Inositol Metabolism (2)
Phospholipid Metabolism (8)
Phosphatidylserine (PS) (2)
Lysophospholipid (7)
Glycerolipid Metabolism (2)
Sphingolipid Synthesis (3)
Ceramide Pes (1)
Sphingosines (2)
Mevalonate Metabolism (1)
Sterol (7)
Pregnenolone Steroids (9)
Progestin Steroids (7)
Corticosteroids (8)
Androgenic Steroids (25)
Estrogenic Steroids (1)
Primary Bile Acid Metabolism (14)
Secondary Bile Acid Metabolism (32)
**NUCLEOTIDE METABOLISM**
Purine Metabolism, (Hypo)Xanthine/Inosine containing (7)
Purine Metabolism, Adenine containing (13)
Pyrimidine Metabolism, Orotate containing (4)
Pyrimidine Metabolism, Uracil containing (12)
Pyrimidine Metabolism, Cytidine containing (7)
Purine and Pyrimidine Metabolism (1)

COFACTORS AND VITAMINS
Nicotinate and Nicotinamide Metabolism (6)
Pantothenate and CoA Metabolism (2)
Ascorbate and Aldarate Metabolism (6)
Tocopherol Metabolism (9)
Hemoglobin and Porphyrin Metabolism (6)
Vitamin A Metabolism (6)
Vitamin B6 Metabolism (1)
**XENOBIOTICS**
Benzoate Metabolism (34)
Xanthine Metabolism (14)
Tobacco Metabolite (6)
Food Component/Plant (74)
Bacterial/Fungal
Bacterial/Fungal
Drug - Analgesics, Anesthetics (29)
Drug - Antibiotic (16)
Drug - Antiviral (1)
Drug - Antiinflammatory, Immunosuppressant (1)
Drug - Antineoplastic (1)
Drug - Cardiovascular (13)
Drug - Gastrointestinal (6)
Drug - Metabolic (4)
Drug - Neurological (15)
Drug - Psychoactive (13)
Drug - Respiratory (3)
Drug - Topical Agents (4)
Drug - Other (2)
Chemical (36)
**PARTIALLY CHARACTERIZED MOLECULES (29)**

Table S3. Metabolic differences of individual metabolites between patients fulfilling the Sepsis-3 definition (PMID 26903338) versus patients only fulfilling the Sepsis-2 criteria (PMID 12682500, 12664219). All individual metabolites showing a significant difference in ANOVA analysis are listed in the figure. The metabolites are listed according to their classification. The table presents from left to right significant correlation with the SOFA score (significance indicated by blue), significantly differing metabolites from the comparions of Gram-positive versus Gram-negative infections (G+/-) and without/with bacteriemia indicated by color and ratio of mean levels, ratio of mean levels (Sepsis-3 level relative to Sepsis-2 level) with corresponding p- and q-values, and the classification/identity of the identified individual metabolite. A ratio >1.00 is indicated by red (i.e. higher in patients with Gran-negative infecton, patients with bacteremia or patients fulfilling the Sepsis-3 criteria) whereas a ratio <1.00 is indicated by green (* The metabolite identity not confirmed based on a standard).

SOFA	G-/+	Without or with bacteremia	SEPSIS-3 versus Sepsis-2	p-value	q-value	SUBPATHWAY	IDENTITY
**AMINO ACIDS AND PEPTIDES**							
	0,72	**0,70**	**1,18**	0,0406	0,3702	Alanine and Aspartate Metabolism	N-acetylalanine
	1,07	1,18	**1,79**	0,0016	0,1304	Alanine and Aspartate Metabolism	N-carbamoylalanine
	1,07	**1,56**	**1,90**	0,0130	0,2556	Glutamate Metabolism	carboxyethyl-GABA
	0,86	0,79	**1,36**	0,0284	0,3266	Histidine Metabolism	1-methylhistidine
	0,92	1,13	**1,87**	0,0135	0,2574	Histidine Metabolism	N-acetyl-1-methylhistidine*
	0,79	1,03	**1,85**	0,0492	0,4051	Histidine Metabolism	imidazole lactate
	0,64	0,70	**1,32**	0,0418	0,3750	Histidine Metabolism	1-methyl-4-imidazoleacetate
	0,39	0,36	**0,57**	0,0290	0,3266	Histidine Metabolism	1-methyl-5-imidazoleacetate
	0,39	0,36	**0,65**	0,0392	0,3702	Histidine Metabolism	1-methyl-5-imidazolelactate
	0,60	0,70	**1,22**	0,0241	0,3221	Lysine Metabolism	5-(galactosylhydroxy)-lysine
	1,07	0,87	**2,33**	0,0180	0,2849	Tyrosine Metabolism	N-acetyltyrosine
	1,02	1,07	**2,22**	0,0073	0,2422	Tyrosine Metabolism	3-(4-hydroxyphenyl)lactate
	2,75	2,63	**3,55**	0,0059	0,2338	Tyrosine Metabolism	phenol sulfate
	0,98	1,02	**1,56**	0,0378	0,3702	Tyrosine Metabolism	N-formylphenylalanine
	0,90	0,86	**1,92**	0,0113	0,2556	Tryptophan Metabolism	kynurenine
	1,30	1,05	**1,61**	0,0257	0,3249	Tryptophan Metabolism	indolelactate
	0,82	0,73	**1,54**	0,0240	0,3221	Leucine, Isoleucine and Valine Metabolism	beta-hydroxyisovaleroylcarnitine
	0,66	0,72	**1,24**	0,0277	0,3249	Leucine, Isoleucine and Valine Metabolism	3-methylglutaconate
	**0,78**	0,93	**0,78**	0,0369	0,3702	Leucine, Isoleucine and Valine Metabolism	isoleucine

	0,85	0,68	1,75	0,0116	0,2556	Leucine, Isoleucine and Valine Metabolism	tiglylcarnitine (C5:1-DC)
	0,90	0,93	1,37	0,0383	0,3702	Leucine, Isoleucine and Valine Metabolism	3-hydroxy-2-ethylpropionate
	0,80	0,75	1,10	0,0100	0,2556	Leucine, Isoleucine and Valine Metabolism	N-carbamoylvaline
	0,31	0,42	0,29	0,0162	0,2703	Methionine, Cysteine, SAM and Taurine Metabolism	N-acetylmethionine sulfoxide
	0,63	0,60	1,23	0,0405	0,3702	Methionine, Cysteine, SAM and Taurine Metabolism	2,3-dihydroxy-5-methylthio-4-pentenoate (DMTPA)*
	0,81	0,74	1,70	0,0393	0,3702	Methionine, Cysteine, SAM and Taurine Metabolism	cysteine
	0,94	0,88	0,83	0,0150	0,2660	Urea cycle; Arginine and Proline Metabolism	arginine
	1,03	1,01	1,96	0,0010	0,1276	Urea cycle; Arginine and Proline Metabolism	urea
	1,13	1,00	1,74	0,0256	0,3249	Urea cycle; Arginine and Proline Metabolism	homocitrulline
	0,47	0,76	1,16	0,0120	0,2556	Urea cycle; Arginine and Proline Metabolism	N2,N5-diacetylornithine
	0,77	1,08	3,56	0,0020	0,1304	Urea cycle; Arginine and Proline Metabolism	argininate*
	0,70	0,83	1,71	0,0485	0,4033	Polyamine Metabolism	(N(1) + N(8))-acetylspermidine
	0,90	0,64	1,57	0,0275	0,3249	Polyamine Metabolism	acisoga
	1,85	1,86	4,54	0,0246	0,3221	Polyamine Metabolism	N1,N12-diacetylspermine
	0,58	0,76	1,71	0,0372	0,3702	Polyamine Metabolism	4-acetamidobutanoate
	0,95	1,43	2,21	0,0015	0,1304	Guanidino and Acetamido Metabolism	guanidinosuccinate
	0,91	0,81	0,80	0,0349	0,3574	Gamma-glutamyl Amino Acid	gamma-glutamylserine
	0,73	0,77	0,65	0,0231	0,3221	Dipeptide	isoleucylglycine
	0,89	0,59	0,59	0,0268	0,3249	Fibrinogen Cleavage Peptide	fibrinopeptide A (3-15)**
	0,91	0,70	0,76	0,0147	0,2660	Fibrinogen Cleavage Peptide	fibrinopeptide B (1-11)**
	0,72	0,75	0,80	0,0089	0,2556	Fibrinogen Cleavage Peptide	fibrinopeptide B (1-12)**
**CARBOHYDRATE**							
	1,06	1,14	1,35	0,0323	0,3485	Glycolysis, Gluconeogenesis, and Pyruvate Metabolism	lactate
	1,11	1,10	1,32	0,0129	0,2556	Pentose Metabolism	ribonate
	0,88	0,78	1,36	0,0232	0,3221	Pentose Metabolism	arabinose
	0,74	0,72	1,45	0,0206	0,3069	Pentose Metabolism	arabitol/xylitol
	2,27	2,08	3,71	0,0059	0,2338	Fructose, Mannose and Galactose Metabolism	galactonate
	0,60	0,65	1,12	0,0346	0,3574	Aminosugar Metabolism	erythronate*
	1,02	0,88	1,32	0,0484	0,4033	Aminosugar Metabolism	N-acetylglucosamine/N-acetylgalactosamine

**ENERGY METABOLISM**							
	0,88	1,12	1,68	0,0168	0,2741	TCA Cycle	alpha-ketoglutarate
	0,97	1,00	1,77	0,0224	0,3221	TCA Cycle	fumarate
**LIPIDS**							
	1,48	1,56	2,13	0,0153	0,2660	Fatty Acid, Amino	N-acetyl-2-aminooctanoate*
	0,90	0,85	0,65	0,0109	0,2556	Fatty Acid Metabolism (Acyl Choline)	palmitoylcholine
	0,82	1,28	0,62	0,0089	0,2556	Fatty Acid Metabolism (Acyl Choline)	dihomo-linolenoyl-choline
	0,95	0,91	0,67	0,0130	0,2556	Fatty Acid Metabolism (Acyl Choline)	linoleoylcholine*
	0,92	0,91	0,65	0,0107	0,2556	Fatty Acid Metabolism (Acyl Choline)	stearoylcholine*
	0,86	1,03	0,60	0,0071	0,2422	Fatty Acid Metabolism (Acyl Choline)	arachidonoylcholine
	0,59	1,38	0,45	0,0467	0,4011	Eicosanoid	12-HETE
	0,84	1,06	0,73	0,0244	0,3221	Endocannabinoid	N-stearoylserine*
	0,62	0,69	1,15	0,0408	0,3702	Mevalonate Metabolism	3-hydroxy-3-methylglutarate
	0,39	1,25	1,58	0,0328	0,3489	Primary Bile Acid Metabolism	glycocholate
**NUCLEOTIDE**							
	1,04	1,04	1,48	0,0003	0,1276	Purine Metabolism, (Hypo)Xanthine/Inosine containing	urate
	1,70	1,74	2,33	0,0020	0,1304	Purine Metabolism, (Hypo)Xanthine/Inosine containing	allantoin
	0,47	0,80	1,49	0,0457	0,3968	Purine Metabolism, Adenine containing	N6-succinyladenosine
	2,79	6,70	4,58	0,0044	0,2066	Pyrimidine Metabolism, Orotate containing	orotidine
	0,84	1,15	1,90	0,0289	0,3266	Pyrimidine Metabolism, Uracil containing	uracil
	0,53	0,63	1,10	0,0481	0,4033	Pyrimidine Metabolism, Uracil containing	3-(3-amino-3-carboxypropyl)uridine*
	1,18	1,14	1,87	0,0062	0,2358	Pyrimidine Metabolism, Thymine containing	5,6-dihydrothymine
**COFACTORS AND VITAMINS**							
	0,83	1,18	2,20	0,0040	0,2054	Nicotinate and Nicotinamide Metabolism	N1-methyl-2-pyridone-5-carboxamide
	0,90	1,02	2,03	0,0206	0,3069	Nicotinate and Nicotinamide Metabolism	N1-methyl-4-pyridone-3-carboxamide
	0,53	1,56	2,20	0,0160	0,2703	Pantothenate and CoA Metabolism	pantoate
	0,99	0,92	1,29	0,0182	0,2849	Ascorbate and Aldarate Metabolism	ascorbic acid 3-sulfate*
	0,72	0,71	1,21	0,0339	0,3564	Ascorbate and Aldarate Metabolism	2-O-methylascorbic acid

	0,55	0,78	1,32	0,0115	0,2556	Vitamin B6 Metabolism	pyridoxate
**XENOBIOTICS**							
	2,17	2,56	3,78	0,0208	0,3069	Food Component/Plant	2,8-quinolinediol sulfate
	1,39	1,10	1,46	0,0068	0,2422	Food Component/Plant	3-formylindole
	0,79	0,84	1,48	0,0090	0,2556	Food Component/Plant	erythritol
	1,11	0,86	2,38	0,0041	0,2054	Food Component/Plant	2-keto-3-deoxy-gluconate
	0,98	1,24	0,46	0,0299	0,3312	Food Component/Plant	tartronate (hydroxymalonate)
	3,95	3,89	5,34	0,0021	0,1304	Food Component/Plant	vanillic acid glycine
	11,97	0,28	0,08	0,0153	0,2660	Drug - Antibiotic	trimethoprim
	5,35	5,08	3,10	0,0056	0,2338	Drug - Cardiovascular	metoprolol
	2,70	2,48	2,36	0,0024	0,1350	Drug - Cardiovascular	metoprolol acid metabolite*
	3,24	3,11	3,26	0,0000	0,0057	Drug - Cardiovascular	alpha-hydroxymetoprolol
	1,12	1,13	1,10	0,0112	0,2556	Drug - Cardiovascular	warfarin
	1,60	1,72	1,55	0,0011	0,1276	Drug - Cardiovascular	6-hydroxywarfarin
	3,65	5,52	5,10	0,0007	0,1276	Drug - Cardiovascular	7-hydroxywarfarin
	2,38	2,53	2,09	0,0011	0,1276	Drug - Cardiovascular	10-hydroxywarfarin
	13,19	2,42	37,65	0,0018	0,1304	Drug - Gastrointestinal	pantoprazole
	1,08	2,35	2,77	0,0273	0,3249	Drug - Topical Agents	2,6-dihydroxybenzoic acid
	0,71	0,76	1,07	0,0131	0,2556	Drug - Topical Agents	hydroquinone sulfate
	1,16	1,20	1,70	0,0010	0,1276	Chemical	sulfate*
	1,41	1,16	1,86	0,0432	0,3837	Chemical	dimethyl sulfone
	1,31	1,57	1,95	0,0449	0,3940	Chemical	triethanolamine
	0,61	0,55	1,93	0,0304	0,3328	Chemical	(2-butoxyethoxy)acetic acid
**PARTIALLY CHARACTERIZED**							
	0,67	0,77	1,13	0,0397	0,3702	Partially Characterized Molecules	glycine conjugate of C10H12O2*
	1,40	1,60	2,18	0,0121	0,2556	Partially Characterized Molecules	bilirubin degradation product, C16H18N2O5 (1)**
	1,40	1,61	2,27	0,0080	0,2556	Partially Characterized Molecules	bilirubin degradation product, C16H18N2O5 (3)**
	1,42	1,61	2,10	0,0268	0,3249	Partially Characterized Molecules	bilirubin degradation product, C16H18N2O5 (4)**

Table S4. Metabolic differences of individual metabolites between patients with and without bacteremia. All individual metabolites showing a significant difference in ANOVA analysis are listed in the figure. The metabolites are listed according to their classification. The table presents from left to right significant correlation with the SOFA score (significance indicated by blue), significantly differing metabolites from the comparions of Gram negative versus Gram positive infections (G+/-) and without/with bacteriemia indicated by color and ratio of mean levels, ratio of mean levels (Sepsis-3 level relative to Sepsis-2 level) with corresponding p- and q-values for the bacteremia comparison, and the classification/identity of the identified individual metabolite. A ratio >1.00 is indicated by red (i.e. higher in patients with bacteremia) whereas a ratio <1.00 is indicated by green (* The metabolite identity not confirmed based on a standard).

SOFA	G-/+	Without or with bacteremia	SEPSIS-3 versus Sepsis-2	p-value	q-value	SUBPATHWAY	IDENTITY
**AMINO ACIDS AND PEPTIDES**							
	0,40	0,36	0,95	0,0209	0,6705	Lysine Metabolism	N,N-dimethyl-5-aminovalerate
	0,55	0,47	2,56	0,0492	0,6705	Tryptophan Metabolism	Picolinate
	1,72	1,66	1,23	0,0177	0,6705	Tryptophan Metabolism	6-bromotryptophan
	0,17	0,11	0,21	0,0234	0,6705	Methionine, Cysteine, SAM and Taurine Metabolism	Cystathionine
	0,91	0,62	1,17	0,0466	0,6705	Methionine, Cysteine, SAM and Taurine Metabolism	alpha-ketobutyrate
	0,54	0,45	0,89	0,0179	0,6705	Urea cycle; Arginine and Proline Metabolism	N,N,N-trimethyl-alanylproline betaine (TMAP)
	0,58	0,55	0,89	0,0217	0,6705	Gamma-glutamyl Amino Acid	gamma-glutamylphenylalanine
	0,68	0,47	0,91	0,0108	0,6115	Gamma-glutamyl Amino Acid	gamma-glutamyl-2-aminobutyrate
	0,91	0,70	0,76	0,0124	0,6115	Fibrinogen Cleavage Peptide	fibrinopeptide B (1-11)**
	0,72	0,75	0,80	0,0034	0,4029	Fibrinogen Cleavage Peptide	fibrinopeptide B (1-12)**
	0,50	0,47	0,85	0,0381	0,6705	Modified Peptides	N,N-dimethyl-pro-pro
**ENERGY**							
	0,74	0,63	0,94	0,0172	0,6705	TCA Cycle	2-methylcitrate/homocitrate
**LIPIDS**							
	0,15	0,08	0,23	0,0122	0,6115	Fatty Acid Synthesis	Malonylcarnitine
	1,24	1,34	1,02	0,0459	0,6705	Short Chain Fatty Acid	butyrate/isobutyrate (4:0)
	0,39	0,43	1,13	0,0490	0,6705	Fatty Acid, Dicarboxylate	decadienedioic acid (C10:2-DC)**
	1,59	1,54	1,23	0,0497	0,6705	Fatty Acid Metabolism (Acyl Carnitine, Monounsaturated)	ximenoylcarnitine (C26:1)*
	0,63	0,55	1,04	0,0452	0,6705	Fatty Acid Metabolism (Acyl Carnitine, Dicarboxylate)	octadecanedioylcarnitine (C18-DC)*
	0,51	0,43	0,96	0,0227	0,6705	Fatty Acid Metabolism (Acyl Carnitine, Dicarboxylate)	octadecenedioylcarnitine (C18:1-DC)*

	0,71	0,62	1,09	0,0252	0,6705	Fatty Acid Metabolism (Acyl Carnitine, Hydroxy)	3-hydroxyoleoylcarnitine
	1,78	2,45	1,68	0,0004	0,0870	Fatty Acid, Monohydroxy	2-hydroxydecanoate
	0,78	0,52	1,03	0,0263	0,6705	Fatty Acid, Monohydroxy	3-hydroxyhexanoate
	1,31	1,94	1,27	0,0196	0,6705	Eicosanoid	leukotriene B4
	0,64	0,40	1,24	0,0342	0,6705	Progestin Steroids	5alpha-pregnan-3beta,20alpha-diol disulfate
	0,53	0,42	0,89	0,0322	0,6705	Progestin Steroids	pregnanediol-3-glucuronide
	0,94	0,19	1,33	0,0093	0,6115	Corticosteroids	cortisol 21-sulfate
	0,51	0,87	1,92	0,0340	0,6705	Primary Bile Acid Metabolism	Taurocholate
	0,75	0,62	1,20	0,0470	0,6705	Secondary Bile Acid Metabolism	taurolithocholate 3-sulfate
	0,24	2,11	4,22	0,0476	0,6705	Secondary Bile Acid Metabolism	taurochenodeoxycholic acid 3-sulfate
**NUCLEOTIDES**							
	0,80	1,62	0,81	0,0263	0,6705	Purine Metabolism, (Hypo)Xanthine/Inosine containing	Xanthine
	1,26	1,46	1,22	0,0251	0,6705	Pyrimidine Metabolism, Uracil containing	5-methyluridine (ribothymidine)
	0,25	0,21	0,33	0,0121	0,6115	Pyrimidine Metabolism, Thymine containing	3-aminoisobutyrate
**COFACTORS AND VITAMINS**							
	1,93	2,50	1,60	0,0431	0,6705	Vitamin A Metabolism	retinol (vitamin A)
**XENOBIOTICS**							
	1,17	0,63	0,77	0,0339	0,6705	Food Component/Plant	methyl glucopyranoside (alpha + beta)
	3,95	3,89	5,34	0,0102	0,6115	Food Component/Plant	vanillic acid glycine
	0,75	0,33	0,72	0,0217	0,6705	Food Component/Plant	4-vinylguaiacol glucuronide
	0,28	0,25	0,55	0,0119	0,6115	Drug - Analgesics, Anesthetics	3-(N-acetyl-cystein-S-yl) acetaminophen
	0,39	0,36	0,46	0,0446	0,6705	Drug - Analgesics, Anesthetics	4-acetamidophenylglucuronide
	0,43	0,48	0,57	0,0438	0,6705	Drug - Analgesics, Anesthetics	2-hydroxyacetaminophen sulfate*
	0,33	0,36	0,40	0,0323	0,6705	Drug - Analgesics, Anesthetics	2-methoxyacetaminophen sulfate*
	0,36	0,33	0,45	0,0460	0,6705	Drug - Analgesics, Anesthetics	2-methoxyacetaminophen glucuronide*
	0,26	0,23	0,46	0,0194	0,6705	Drug - Analgesics, Anesthetics	3-(cystein-S-yl)acetaminophen*
	0,62	0,45	0,49	0,0292	0,6705	Drug - Analgesics, Anesthetics	3-(methylthio)acetaminophen sulfate*
	5,35	5,08	3,10	0,0045	0,4714	Drug - Cardiovascular	Metoprolol
	2,70	2,48	2,36	0,0081	0,6115	Drug - Cardiovascular	metoprolol acid metabolite*

	3,24	3,11	3,26	0,0000	0,0407	Drug - Cardiovascular	alpha-hydroxymetoprolol
	1,12	1,13	1,10	0,0019	0,2707	Drug - Cardiovascular	Warfarin
	1,60	1,72	1,55	0,0002	0,0690	Drug - Cardiovascular	6-hydroxywarfarin
	3,65	5,52	5,10	0,0006	0,0954	Drug - Cardiovascular	7-hydroxywarfarin
	2,38	2,53	2,09	0,0003	0,0802	Drug - Cardiovascular	10-hydroxywarfarin
	13,19	2,42	37,65	0,0446	0,6705	Drug - Gastrointestinal	Pantoprazole
	0,60	0,63	0,88	0,0101	0,6115	Chemical	2-acrylamidoglycolic acid

Table S5. Differences of individual metabolites between patients with Gram-negative versus Gram-positive infection. All individual metabolites showing a significant difference in ANOVA analysis are listed in the figure. The metabolites are listed according to their classification. The table presents from left to right significant correlation with the SOFA score (significance indicated by blue), significantly differing metabolites from the comparions of Gram-positive versus Gram-negative infections (G+/-) and patients without/with bacteriemia indicated by color and ratio of mean levels, ratio of mean levels (corresponding p- and q-values for the G-/+ comparison, and the classification/identity of the identified individual metabolite. A ratio >1.00 is indicated by red (i.e. higher in patients with bacteremia) whereas a ratio <1.00 is indicated by green (* The metabolite identity not confirmed based on a standard).

SOFA	G-/+	Withour or with bacteremia	Sepsis-3 versus Sepsis-2	p-value	q-value	SUBPATHWAY	IDENTITY
**AMINO ACIDS AND PEPTIDES**							
	1,27	1,21	1,09	0,0412	0,9840	Tryptophan Metabolism	tryptophan
	1,72	1,66	1,23	0,0015	0,2685	Tryptophan Metabolism	6-bromotryptophan
	0,71	0,74	0,96	0,0420	0,9840	Leucine, Isoleucine and Valine Metabolism	N-acetylisoleucine
	0,31	0,42	0,29	0,0265	0,9840	Methionine, Cysteine, SAM and Taurine Metabolism	N-acetylmethionine sulfoxide
	1,37	1,04	0,92	0,0360	0,9840	Fibrinogen Cleavage Peptide	fibrinopeptide A (5-16)*
	1,77	1,23	0,94	0,0089	0,7439	Fibrinogen Cleavage Peptide	fibrinopeptide A (7-16)*
	2,17	1,19	0,79	0,0221	0,9840	Fibrinogen Cleavage Peptide	fibrinopeptide A (8-16)**
	0,72	0,75	0,80	0,0097	0,7442	Fibrinogen Cleavage Peptide	fibrinopeptide B (1-12)**
**CARBOHYDRATE**							
	1,30	1,20	1,28	0,0193	0,9840	Glycolysis, Gluconeogenesis, and Pyruvate Metabolism	glucose
	0,05	0,58	0,71	0,0253	0,9840	Disaccharides and Oligosaccharides	sucrose
	0,12	0,40	0,74	0,0472	0,9840	Fructose, Mannose and Galactose Metabolism	mannitol/sorbitol
	2,27	2,08	3,71	0,0138	0,8220	Fructose, Mannose and Galactose Metabolism	galactonate
**LIPIDS**							
	1,24	1,34	1,02	0,0280	0,9840	Short Chain Fatty Acid	butyrate/isobutyrate (4:0)
	0,36	0,84	0,60	0,0138	0,8220	Long Chain Polyunsaturated Fatty Acid (n3 and n6)	mead acid (20:3n9)
	0,30	0,39	0,69	0,0342	0,9840	Fatty Acid, Dicarboxylate	adipate (C6-DC)
	2,22	1,18	1,13	0,0233	0,9840	Fatty Acid, Dicarboxylate	hydroxy-CMPF*
	0,55	0,53	1,01	0,0486	0,9840	Fatty Acid Metabolism (also BCAA Metabolism)	propionylglycine
	0,47	0,68	0,95	0,0087	0,7439	Fatty Acid Metabolism (Acyl Carnitine,	3-decenoylcarnitine

						Monounsaturated)	
	1,75	1,11	0,98	0,0345	0,9840	Fatty Acid Metabolism (Acyl Carnitine, Polyunsaturated)	docosahexaenoylcarnitine (C22:6)*
	0,45	0,99	1,22	0,0479	0,9840	Androgenic Steroids	16alpha-hydroxy DHEA 3-sulfate
	0,46	0,80	0,92	0,0447	0,9840	Androgenic Steroids	andro steroid monosulfate C19H28O6S (1)*
	1,72	0,69	1,11	0,0419	0,9840	Secondary Bile Acid Metabolism	glycodeoxycholate
**XENOBIOTICS**							
	2,17	2,56	3,78	0,0140	0,8220	Food Component/Plant	2,8-quinolinediol sulfate
	0,23	0,34	0,44	0,0404	0,9840	Food Component/Plant	2-oxindole-3-acetate
	1,39	1,10	1,46	0,0115	0,8220	Food Component/Plant	3-formylindole
	3,95	3,89	5,34	0,0217	0,9840	Food Component/Plant	vanillic acid glycine
	0,08	0,41	0,97	0,0211	0,9840	Drug - Analgesics, Anesthetics	lidocaine
	0,05	0,38	1,01	0,0251	0,9840	Drug - Analgesics, Anesthetics	N-ethylglycinexylidide
	11,97	0,28	0,08	0,0153	0,8499	Drug - Antibiotic	trimethoprim
	5,35	5,08	3,10	0,0015	0,2685	Drug - Cardiovascular	metoprolol
	2,70	2,48	2,36	0,0016	0,2685	Drug - Cardiovascular	metoprolol acid metabolite*
	3,24	3,11	3,26	0,0000	0,0123	Drug - Cardiovascular	alpha-hydroxymetoprolol
	1,12	1,13	1,10	0,0061	0,6072	Drug - Cardiovascular	warfarin
	1,60	1,72	1,55	0,0010	0,2685	Drug - Cardiovascular	6-hydroxywarfarin
	3,65	5,52	5,10	0,0020	0,2808	Drug - Cardiovascular	7-hydroxywarfarin
	2,38	2,53	2,09	0,0007	0,2685	Drug - Cardiovascular	10-hydroxywarfarin
	13,19	2,42	37,65	0,0048	0,5797	Drug - Gastrointestinal	pantoprazole
	0,47	0,55	0,69	0,0315	0,9840	Drug - Respiratory	De(carboxymethoxy) cetirizine acetic acid
	0,60	0,63	0,88	0,0052	0,5797	Chemical	2-acrylamidoglycolic acid

Table S6. Summary of individual metabolites between patients with Gram negative and Gram positive infection. All individual metabolites showing a significant difference in ANOVA analysis are listed. The metabolites are listed according to their classification. A ratio of mean levels (Gram-negative relative to Gram-positive infection) <1.00 is indicated by green (i.e. lower in Gram-negative infection) (* The metabolite identity not confirmed based on a standard).

SUBPATHWAY	IDENTITY
**AMINO ACIDS AND PEPTIDES (6/8 associated with SOFA score)**	
Tryptophan Metabolism	Tryptophan, 6-bromotryptophan
Leucine, Isoleucine and Valine Metabolism	**N-acetylisoleucine**
Methionine, Cysteine, SAM and Taurine Metabolism	**N-acetylmethionine sulfoxide**
Fibrinogen Cleavage Peptide	Fibrinopeptide A (5-16)*, fibrinopeptide A (7-16)*, fibrinopeptide A (8-16)**, **fibrinopeptide B (1-12)****
**CARBOHYDRATE (2/4 associated with SOFA score)**	
Glycolysis, Gluconeogenesis, and Pyruvate Metabolism	Glucose
Disaccharides and Oligosaccharides	Sucrose
Fructose, Mannose and Galactose Metabolism	Mannitol/sorbitol
Fructose, Mannose and Galactose Metabolism	Galactonate
**LIPIDS (6/10 associated with SOFA score)**	
Short Chain Fatty Acid	Butyrate/isobutyrate (4:0)
Long Chain Polyunsaturated Fatty Acid (n3 and n6)	**Mead acid (20:3n9)**
Fatty Acid, Dicarboxylate	**Adipate (C6-DC),** hydroxy-CMPF*
Fatty Acid Metabolism (also BCAA Metabolism)	**Propionylglycine**
Fatty Acid Metabolism (Acyl Carnitine, Monounsaturated)	**3-decenoylcarnitine**
Fatty Acid Metabolism (Acyl Carnitine, Polyunsaturated)	Cocosahexaenoylcarnitine (C22:6)*
Androgenic Steroids	16alpha-hydroxy DHEA 3-sulfate, **andro steroid monosulfate C19H28O6S (1)***
Secondary Bile Acid Metabolism	Glycodeoxycholate
**XENOBIOTICS (5/17 associated with SOFA score)**	
Food Component/Plant	2,8-quinolinediol sulfate, **2-oxindole-3-acetate,** 3-formylindole, vanillic acid glycine
Drug - Analgesics, Anesthetics	**Lidocaine, N-ethylglycinexylidide**
Drug - Antibiotic	Trimethoprim
Drug - Cardiovascular	Metoprolol, metoprolol acid metabolite*, alpha-hydroxymetoprolol, warfarin, 6-hydroxywarfarin, 7-hydroxywarfarin, 10-hydroxywarfarin
Drug - Gastrointestinal	Pantoprazole
Drug - Respiratory	**De(carboxymethoxy) cetirizine acetic acid**
Chemical	**2-acrylamidoglycolic acid**

Table S7. Subclassification of the 42 metabolites showing the strongest correlation with total SOFA score, i.e. a p-value <0.05 and a correlation factor >0.6 when using the Pearson test. All metabolites showed a Pearson's q value <0.00005. The metabolites are listed accoring to their position from left to right in the unsupervised hierarchical clustering analysis based on the serum level of these 42 metabolites. The classification of individual metabolites according to the main metabolic pathway is indicated by a color code in the left color. More detailed presentation of the results from the Pearson's test for individual metaboltes are included in Table S8. Based on the metabolite clustering the individual metabolites were classified into four main subset, and the separation of individual metabolite clusters is indicated by bright green color in the left column (* The metabolite identity not confirmed based on a standard).

Main pathway	Sub Pathway	Biochemical Name	Position/ranging according to the cluster presented in Figure 1.	Main metabolite cluster/subset
Amino Acid	Tyrosine Metabolism	3-(4-hydroxyphenyl)lactate	1	A (left)
Amino Acid	Phenylalanine Metabolism	phenyllactate (PLA)	2	
Amino Acid	Methionine, Cysteine, SAM and Taurine Metabolism	2-hydroxy-4-(methylthio)butanoic acid	3	
Amino Acid	Polyamine Metabolism	(N(1) + N(8))-acetylspermidine	4	
Amino Acid	Lysine Metabolism	N6-acetyllysine	5	
Nucleotide	Purine Metabolism, Adenine containing	N1-methyladenosine	6	
Nucleotide	Purine Metabolism, Guanine containing	7-methylguanine	7	
Xenobiotics	Chemical	sulfate*	8	
Amino Acid	Lysine Metabolism	N6,N6,N6-trimethyllysine	9	
Lipid	Fatty Acid, Dicarboxylate	2-hydroxyglutarate	10	
Nucleotide	Pyrimidine Metabolism, Uracil containing	Uracil	11	
Lipid	Fatty Acid, Dihydroxy	2S,3R-dihydroxybutyrate	12	
Amino Acid	Urea cycle; Arginine and Proline Metabolism	Urea	13	
Lipid	Progestin Steroids	5alpha-pregnan-3beta,20beta-diol monosulfate (1)	14	B (left middle)
Lipid	Progestin Steroids	pregnanolone/allopregnanolone sulfate	15	
Amino Acid	Leucine, Isoleucine and Valine Metabolism	Isovalerylglycine	16	
Amino Acid	Leucine, Isoleucine and Valine Metabolism	2-methylbutyrylglycine	17	
Energy	TCA Cycle	succinylcarnitine (C4-DC)	18	
Amino Acid	Lysine Metabolism	glutarylcarnitine (C5-DC)	19	
Amino Acid	Tryptophan Metabolism	xanthurenate	20	

Amino Acid	Tryptophan Metabolism	N-acetylkynurenine (2)	21	**C (right middle)**
Lipid	Fatty Acid Metabolism (Acyl Glycine)	picolinoylglycine	22	
Amino Acid	Tryptophan Metabolism	picolinate	23	
Amino Acid	Tyrosine Metabolism	N-acetyltyrosine	24	
Amino Acid	Phenylalanine Metabolism	N-acetylphenylalanine	25	
Amino Acid	Alanine and Aspartate Metabolism	N-acetylaspartate (NAA)	26	
Amino Acid	Glutamate Metabolism	N-acetylglutamate	27	
Amino Acid	Glycine, Serine and Threonine Metabolism	N-acetylthreonine	28	
Amino Acid	Polyamine Metabolism	N-acetyl-isoputreanine	29	
Xenobiotics	Food Component/Plant	erythritol	30	
Amino Acid	Methionine, Cysteine, SAM and Taurine Metabolism	N-acetyltaurine	31	
Nucleotide	Pyrimidine Metabolism, Cytidine containing	N4-acetylcytidine	32	
Amino Acid	Polyamine Metabolism	N-acetylputrescine	33	
Amino Acid	Tyrosine Metabolism	vanillylmandelate (VMA)	34	
Amino Acid	Tryptophan Metabolism	kynurenate	35	
Lipid	Inositol Metabolism	myo-inositol	36	**D (right)**
Lipid	Primary Bile Acid Metabolism	taurochenodeoxycholate	37	
Lipid	Progestin Steroids	5alpha-pregnan-3beta-ol,20-one sulfate	38	
Lipid	Progestin Steroids	5alpha-pregnan-diol disulfate	39	
Lipid	Pregnenolone Steroids	21-hydroxypregnenolone disulfate	40	
Lipid	Pregnenolone Steroids	pregnenediol disulfate (C21H34O8S2)*	41	
Lipid	Secondary Bile Acid Metabolism	taurocholenate sulfate*	42	

Table S8. The correlation between the serum level of individual metabolites and the total SOFA score; a list presenting all metabolites that showed a p-value corresponding to <0.05 when using the Pearson's correlation test. The table presents the main metabolic pathway, the subpathway, the identity of the metabolite, and the Pearson p. q and the correlation values. The correlation values are presented as the absolute values, and negative valies are marked with green color. The classification of each metabolite with regard to the main metabolic pathway is indicated by a color code in the left column for metabolites showing an absolute correlation value exceeding 0.5 (* The metabolite identity not confirmed based on a standard).

Main pathway	Sub Pathway	Biochemical Name	Pearson p	Pearson q	Pearson correlation
Amino Acid	Tyrosine Metabolism	3-(4-hydroxyphenyl)lactate	0,0000	0,0000	0,7805
Amino Acid	Phenylalanine Metabolism	phenyllactate (PLA)	0,0000	0,0000	0,7061
Lipid	Progestin Steroids	5alpha-pregnan-3beta,20beta-diol monosulfate (1)	0,0000	0,0000	0,6905
Amino Acid	Tryptophan Metabolism	N-acetylkynurenine (2)	0,0000	0,0000	0,6883
Amino Acid	Leucine, Isoleucine and Valine Metabolism	isovalerylglycine	0,0000	0,0000	0,6793
Lipid	Secondary Bile Acid Metabolism	taurocholenate sulfate*	0,0000	0,0000	0,6763
Energy	TCA Cycle	succinylcarnitine (C4-DC)	0,0000	0,0000	0,6734
Amino Acid	Tryptophan Metabolism	picolinate	0,0000	0,0000	0,6712
Lipid	Progestin Steroids	5alpha-pregnan-3beta-ol,20-one sulfate	0,0000	0,0000	0,6636
Amino Acid	Lysine Metabolism	N6-acetyllysine	0,0000	0,0000	0,6611
Lipid	Fatty Acid Metabolism (Acyl Glycine)	picolinoylglycine	0,0000	0,0000	0,6596
Nucleotide	Pyrimidine Metabolism, Uracil containing	uracil	0,0000	0,0000	0,6595
Lipid	Progestin Steroids	pregnanolone/allopregnanolone sulfate	0,0000	0,0000	0,6579
Lipid	Fatty Acid, Dihydroxy	2S,3R-dihydroxybutyrate	0,0000	0,0000	0,6506
Amino Acid	Tyrosine Metabolism	N-acetyltyrosine	0,0000	0,0000	0,6487
Nucleotide	Purine Metabolism, Guanine containing	7-methylguanine	0,0000	0,0000	0,6439
Amino Acid	Urea cycle; Arginine and Proline Metabolism	urea	0,0000	0,0000	0,6432
Amino Acid	Lysine Metabolism	N6,N6,N6-trimethyllysine	0,0000	0,0000	0,6426
Amino Acid	Tyrosine Metabolism	vanillylmandelate (VMA)	0,0000	0,0000	0,6425
Amino Acid	Polyamine Metabolism	N-acetylputrescine	0,0000	0,0000	0,6404
Lipid	Inositol Metabolism	myo-inositol	0,0000	0,0000	0,6376
Amino Acid	Leucine, Isoleucine and Valine Metabolism	2-methylbutyrylglycine	0,0000	0,0000	0,6360
Amino Acid	Polyamine Metabolism	N-acetyl-isoputreanine	0,0000	0,0000	0,6338
Amino Acid	Polyamine Metabolism	(N(1) + N(8))-acetylspermidine	0,0000	0,0000	0,6333

Amino Acid	Alanine and Aspartate Metabolism	N-acetylaspartate (NAA)	0,0000	0,0000	0,6330
Lipid	Progestin Steroids	5alpha-pregnan-diol disulfate	0,0000	0,0000	0,6320
Amino Acid	Methionine, Cysteine, SAM and Taurine Metabolism	2-hydroxy-4-(methylthio)butanoic acid	0,0000	0,0000	0,6315
Amino Acid	Tryptophan Metabolism	kynurenate	0,0000	0,0000	0,6284
Amino Acid	Lysine Metabolism	glutarylcarnitine (C5-DC)	0,0000	0,0000	0,6264
Lipid	Pregnenolone Steroids	pregnenediol disulfate (C21H34O8S2)*	0,0000	0,0000	0,6248
Lipid	Pregnenolone Steroids	21-hydroxypregnenolone disulfate	0,0000	0,0000	0,6244
Amino Acid	Glycine, Serine and Threonine Metabolism	N-acetylthreonine	0,0000	0,0000	0,6220
Amino Acid	Phenylalanine Metabolism	N-acetylphenylalanine	0,0000	0,0000	0,6178
Nucleotide	Purine Metabolism, Adenine containing	N1-methyladenosine	0,0000	0,0000	0,6145
Amino Acid	Tryptophan Metabolism	xanthurenate	0,0000	0,0000	0,6139
Lipid	Fatty Acid, Dicarboxylate	2-hydroxyglutarate	0,0000	0,0000	0,6134
Nucleotide	Pyrimidine Metabolism, Cytidine containing	N4-acetylcytidine	0,0000	0,0000	0,6125
Lipid	Primary Bile Acid Metabolism	taurochenodeoxycholate	0,0000	0,0000	0,6078
Xenobiotics	Chemical	sulfate*	0,0000	0,0000	0,6072
Amino Acid	Methionine, Cysteine, SAM and Taurine Metabolism	N-acetyltaurine	0,0000	0,0000	0,6067
Xenobiotics	Food Component/Plant	erythritol	0,0000	0,0000	0,6017
Amino Acid	Glutamate Metabolism	N-acetylglutamate	0,0000	0,0000	0,6010
Amino Acid	Polyamine Metabolism	4-acetamidobutanoate	0,0000	0,0000	0,5971
Carbohydrate	Pentose Metabolism	arabitol/xylitol	0,0000	0,0000	0,5928
Xenobiotics	Chemical	2-methoxyresorcinol sulfate	0,0000	0,0000	0,5919
Nucleotide	Purine Metabolism, Adenine containing	adenosine 3',5'-cyclic monophosphate (cAMP)	0,0000	0,0000	0,5916
Amino Acid	Leucine, Isoleucine and Valine Metabolism	tiglylcarnitine (C5:1-DC)	0,0000	0,0000	0,5910
Amino Acid	Urea cycle; Arginine and Proline Metabolism	argininate*	0,0000	0,0000	0,5904
Cofactors and Vitamins	Nicotinate and Nicotinamide Metabolism	1-methylnicotinamide	0,0000	0,0000	0,5882
Amino Acid	Histidine Metabolism	N-acetylhistidine	0,0000	0,0000	0,5864
Lipid	Pregnenolone Steroids	pregnenetriol disulfate*	0,0000	0,0000	0,5848
Carbohydrate	Aminosugar Metabolism	N-acetylneuraminate	0,0000	0,0000	0,5829

Amino Acid	Tryptophan Metabolism	indoleacetylglutamine	0,0000	0,0000	0,5813
Amino Acid	Alanine and Aspartate Metabolism	N-acetylalanine	0,0000	0,0000	0,5786
Carbohydrate	Aminosugar Metabolism	N-acetylglucosaminylasparagine	0,0000	0,0000	0,5784
Nucleotide	Pyrimidine Metabolism, Uracil containing	3-(3-amino-3-carboxypropyl)uridine*	0,0000	0,0000	0,5776
Amino Acid	Leucine, Isoleucine and Valine Metabolism	2,3-dihydroxy-2-methylbutyrate	0,0000	0,0000	0,5764
Lipid	Androgenic Steroids	5alpha-androstan-3alpha,17alpha-diol disulfate	0,0000	0,0000	0,5751
Amino Acid	Methionine, Cysteine, SAM and Taurine Metabolism	2,3-dihydroxy-5-methylthio-4-pentenoate (DMTPA)*	0,0000	0,0000	0,5733
Amino Acid	Glycine, Serine and Threonine Metabolism	sarcosine	0,0000	0,0000	0,5732
Lipid	Carnitine Metabolism	deoxycarnitine	0,0000	0,0000	0,5726
Cofactors and Vitamins	Nicotinate and Nicotinamide Metabolism	quinolinate	0,0000	0,0000	0,5675
Amino Acid	Polyamine Metabolism	N1,N12-diacetylspermine	0,0000	0,0000	0,5670
Lipid	Pregnenolone Steroids	pregnenolone sulfate	0,0000	0,0000	0,5643
Xenobiotics	Bacterial/Fungal	N-methylpipecolate	0,0000	0,0000	0,5636
Lipid	Primary Bile Acid Metabolism	glycocholate	0,0000	0,0000	0,5626
Lipid	Secondary Bile Acid Metabolism	taurochenodeoxycholic acid 3-sulfate	0,0000	0,0000	0,5623
Amino Acid	Leucine, Isoleucine and Valine Metabolism	beta-hydroxyisovaleroylcarnitine	0,0000	0,0000	0,5610
Nucleotide	Pyrimidine Metabolism, Uracil containing	5,6-dihydrouridine	0,0000	0,0000	0,5602
Nucleotide	Pyrimidine Metabolism, Uracil containing	pseudouridine	0,0000	0,0000	0,5595
Lipid	Pregnenolone Steroids	pregnenediol sulfate (C21H34O5S)*	0,0000	0,0000	0,5592
Amino Acid	Lysine Metabolism	N-acetyl-2-aminoadipate	0,0000	0,0000	0,5575
Amino Acid	Leucine, Isoleucine and Valine Metabolism	N-acetylvaline	0,0000	0,0000	0,5558
Amino Acid	Creatine Metabolism	creatine	0,0000	0,0000	0,5549
Amino Acid	Histidine Metabolism	formiminoglutamate	0,0000	0,0000	0,5543
Carbohydrate	Aminosugar Metabolism	N-acetylglucosamine/N-acetylgalactosamine	0,0000	0,0000	0,5520
Amino Acid	Phenylalanine Metabolism	phenylpyruvate	0,0000	0,0000	0,5518
Amino Acid	Lysine Metabolism	hydroxy-N6,N6,N6-trimethyllysine*	0,0000	0,0000	0,5512
Lipid	Androgenic Steroids	androstenediol (3beta,17beta) disulfate (2)	0,0000	0,0000	0,5510
Lipid	Secondary Bile Acid Metabolism	glycoursodeoxycholic acid sulfate (2)	0,0000	0,0000	0,5500
Amino Acid	Leucine, Isoleucine and Valine Metabolism	methylsuccinate	0,0000	0,0000	0,5467

Lipid	Secondary Bile Acid Metabolism	taurolithocholate 3-sulfate	0,0000	0,0000	0,5420
Amino Acid	Leucine, Isoleucine and Valine Metabolism	isobutyrylglycine	0,0000	0,0000	0,5417
Nucleotide	Pyrimidine Metabolism, Uracil containing	N-acetyl-beta-alanine	0,0000	0,0000	0,5417
Lipid	Primary Bile Acid Metabolism	glycochenodeoxycholate 3-sulfate	0,0000	0,0000	0,5416
Lipid	Pregnenolone Steroids	pregnenetriol sulfate*	0,0000	0,0000	0,5409
Lipid	Primary Bile Acid Metabolism	glycochenodeoxycholate	0,0000	0,0000	0,5409
Nucleotide	Purine Metabolism, Adenine containing	N6-succinyladenosine	0,0000	0,0000	0,5409
Amino Acid	Glycine, Serine and Threonine Metabolism	N-acetylserine	0,0000	0,0000	0,5393
Lipid	Fatty Acid, Dicarboxylate	decadienedioic acid (C10:2-DC)**	0,0000	0,0000	0,5393
Amino Acid	Tyrosine Metabolism	4-hydroxyphenylpyruvate	0,0000	0,0000	0,5388
Amino Acid	Glutamate Metabolism	N-acetyl-aspartyl-glutamate (NAAG)	0,0000	0,0000	0,5387
Lipid	Secondary Bile Acid Metabolism	glycolithocholate sulfate*	0,0000	0,0000	0,5385
Amino Acid	Tryptophan Metabolism	5-hydroxyindoleacetate	0,0000	0,0000	0,5384
Amino Acid	Creatine Metabolism	creatinine	0,0000	0,0000	0,5371
Energy	TCA Cycle	2-methylcitrate/homocitrate	0,0000	0,0000	0,5351
Amino Acid	Histidine Metabolism	N-acetylcarnosine	0,0000	0,0000	0,5347
Carbohydrate	Aminosugar Metabolism	erythronate*	0,0000	0,0000	0,5337
Amino Acid	Histidine Metabolism	1-methyl-4-imidazoleacetate	0,0000	0,0000	0,5333
Carbohydrate	Aminosugar Metabolism	glucuronate	0,0000	0,0000	0,5329
Amino Acid	Leucine, Isoleucine and Valine Metabolism	3-methylglutaconate	0,0000	0,0000	0,5316
Lipid	Progestin Steroids	5alpha-pregnan-3beta,20alpha-diol monosulfate (2)	0,0000	0,0001	0,5298
Peptide	Gamma-glutamyl Amino Acid	gamma-glutamyl-epsilon-lysine	0,0000	0,0001	0,5273
Lipid	Fatty Acid Metabolism (Acyl Carnitine, Medium Chain)	hexanoylcarnitine (C6)	0,0000	0,0001	0,5266
Amino Acid	Tryptophan Metabolism	indole-3-carboxylate	0,0000	0,0001	0,5262
Cofactors and Vitamins	Ascorbate and Aldarate Metabolism	2-O-methylascorbic acid	0,0000	0,0001	0,5232
Lipid	Secondary Bile Acid Metabolism	glycodeoxycholate 3-sulfate	0,0000	0,0001	0,5221
Cofactors and Vitamins	Tocopherol Metabolism	alpha-CEHC sulfate	0,0000	0,0001	0,5218
Lipid	Fatty Acid, Dihydroxy	2R,3R-dihydroxybutyrate	0,0000	0,0001	0,5207
Amino Acid	Leucine, Isoleucine and Valine Metabolism	tigloylglycine	0,0000	0,0001	0,5160

Amino Acid	Glutamate Metabolism	N-acetylglutamine	0,0000	0,0001	0,5158
Amino Acid	Tryptophan Metabolism	8-methoxykynurenate	0,0000	0,0001	0,5157
Lipid	Secondary Bile Acid Metabolism	taurohyocholate*	0,0000	0,0001	0,5147
Lipid	Secondary Bile Acid Metabolism	taurodeoxycholic acid 3-sulfate	0,0000	0,0001	0,5146
Amino Acid	Urea cycle; Arginine and Proline Metabolism	2-oxoarginine*	0,0000	0,0001	0,5137
Amino Acid	Methionine, Cysteine, SAM and Taurine Metabolism	N-formylmethionine	0,0000	0,0001	0,5136
Lipid	Primary Bile Acid Metabolism	glycochenodeoxycholate glucuronide (1)	0,0000	0,0001	0,5130
Lipid	Fatty Acid Metabolism (Acyl Carnitine, Hydroxy)	3-hydroxyoctanoylcarnitine (1)	0,0000	0,0001	0,5120
Lipid	Primary Bile Acid Metabolism	glycocholate sulfate	0,0000	0,0001	0,5114
Nucleotide	Purine Metabolism, Guanine containing	N2,N2-dimethylguanosine	0,0000	0,0001	0,5102
Amino Acid	Tryptophan Metabolism	kynurenine	0,0000	0,0001	0,5093
Lipid	Fatty Acid, Dicarboxylate	tetradecadienedioate (C14:2-DC)*	0,0000	0,0001	0,5082
Nucleotide	Purine Metabolism, Adenine containing	N6-carbamoylthreonyladenosine	0,0000	0,0001	0,5081
Amino Acid	Methionine, Cysteine, SAM and Taurine Metabolism	5-methylthioribose**	0,0000	0,0001	0,5078
Amino Acid	Histidine Metabolism	1-methylhistidine	0,0000	0,0001	0,5072
Lipid	Phospholipid Metabolism	glycerophosphorylcholine (GPC)	0,0000	0,0001	0,5069
Lipid	Sphingosines	sphingosine 1-phosphate	0,0000	0,0001	0,5067
Lipid	Secondary Bile Acid Metabolism	glycohyocholate	0,0000	0,0001	0,5023
Amino Acid	Tyrosine Metabolism	thyroxine	0,0000	0,0001	0,5020
Peptide	Acetylated Peptides	phenylacetylglutamate	0,0000	0,0001	0,5008
Lipid	Lysophospholipid	1-palmitoyl-GPA (16:0)	0,0000	0,0001	0,5006
Lipid	Secondary Bile Acid Metabolism	glycocholenate sulfate*	0,0000	0,0001	0,4994
Amino Acid	Histidine Metabolism	N-acetyl-1-methylhistidine*	0,0000	0,0001	0,4991
Xenobiotics	Tobacco Metabolite	3-hydroxycotinine glucuronide	0,0000	0,0001	0,4991
Lipid	Fatty Acid, Dicarboxylate	maleate	0,0001	0,0002	0,4984
Amino Acid	Urea cycle; Arginine and Proline Metabolism	N-acetylcitrulline	0,0001	0,0002	0,4982
Nucleotide	Purine Metabolism, (Hypo)Xanthine/Inosine containing	N1-methylinosine	0,0001	0,0002	0,4957
Amino Acid	Histidine Metabolism	imidazole lactate	0,0001	0,0002	0,4956
Lipid	Primary Bile Acid Metabolism	taurocholate	0,0001	0,0002	0,4954

Peptide	Gamma-glutamyl Amino Acid	gamma-glutamylhistidine	0,0001	0,0002	0,4946
Amino Acid	Guanidino and Acetamido Metabolism	guanidinosuccinate	0,0001	0,0002	0,4937
Nucleotide	Pyrimidine Metabolism, Uracil containing	5,6-dihydrouracil	0,0001	0,0002	0,4928
Carbohydrate	Fructose, Mannose and Galactose Metabolism	galactonate	0,0001	0,0002	0,4924
Lipid	Corticosteroids	tetrahydrocortisol sulfate (1)	0,0001	0,0002	0,4908
Xenobiotics	Benzoate Metabolism	4-hydroxybenzoate	0,0001	0,0002	0,4873
Lipid	Progestin Steroids	5alpha-pregnan-3beta,20alpha-diol disulfate	0,0001	0,0002	0,4870
Amino Acid	Tryptophan Metabolism	indolelactate	0,0001	0,0002	0,4863
Amino Acid	Lysine Metabolism	5-(galactosylhydroxy)-lysine	0,0001	0,0002	0,4855
Xenobiotics	Chemical	trizma acetate	0,0001	0,0002	0,4854
Xenobiotics	Benzoate Metabolism	2-ethylphenylsulfate	0,0001	0,0002	0,4850
Nucleotide	Pyrimidine Metabolism, Thymine containing	5,6-dihydrothymine	0,0001	0,0002	0,4841
Lipid	Fatty Acid Metabolism (Acyl Carnitine, Dicarboxylate)	adipoylcarnitine (C6-DC)	0,0001	0,0002	0,4839
Lipid	Progestin Steroids	pregnanediol-3-glucuronide	0,0001	0,0002	0,4839
Xenobiotics	Drug - Analgesics, Anesthetics	methadone	0,0001	0,0002	0,4839
Lipid	Fatty Acid, Dicarboxylate	hexadecenedioate (C16:1-DC)*	0,0001	0,0002	0,4834
Xenobiotics	Food Component/Plant	2-keto-3-deoxy-gluconate	0,0001	0,0003	0,4802
Amino Acid	Histidine Metabolism	1-ribosyl-imidazoleacetate*	0,0001	0,0003	0,4784
Amino Acid	Glutamate Metabolism	carboxyethyl-GABA	0,0001	0,0003	0,4775
Amino Acid	Leucine, Isoleucine and Valine Metabolism	isovalerylcarnitine (C5)	0,0001	0,0003	0,4770
Amino Acid	Urea cycle; Arginine and Proline Metabolism	N-acetylproline	0,0001	0,0003	0,4768
Nucleotide	Purine Metabolism, (Hypo)Xanthine/Inosine containing	hypoxanthine	0,0001	0,0003	0,4762
Nucleotide	Purine Metabolism, Adenine containing	N6-methyladenosine	0,0001	0,0003	0,4749
Lipid	Fatty Acid Metabolism (Acyl Carnitine, Hydroxy)	3-hydroxydecanoylcarnitine	0,0001	0,0003	0,4735
Amino Acid	Tryptophan Metabolism	C-glycosyltryptophan	0,0001	0,0003	0,4722
Lipid	Mevalonate Metabolism	3-hydroxy-3-methylglutarate	0,0001	0,0003	0,4721
Amino Acid	Leucine, Isoleucine and Valine Metabolism	isobutyrylcarnitine (C4)	0,0001	0,0003	0,4716
Cofactors and Vitamins	Tocopherol Metabolism	alpha-CMBHC glucuronide	0,0001	0,0003	0,4712
Xenobiotics	Benzoate Metabolism	3-methoxycatechol sulfate (1)	0,0001	0,0004	0,4709

Amino Acid	Polyamine Metabolism	5-methylthioadenosine (MTA)	0,0002	0,0004	0,4693
Lipid	Secondary Bile Acid Metabolism	isoursodeoxycholate sulfate (1)	0,0002	0,0004	0,4689
Amino Acid	Polyamine Metabolism	acisoga	0,0002	0,0004	0,4688
Lipid	Secondary Bile Acid Metabolism	glycoursodeoxycholic acid sulfate (1)	0,0002	0,0004	0,4681
Xenobiotics	Chemical	2-acrylamidoglycolic acid	0,0002	0,0004	0,4670
Amino Acid	Tyrosine Metabolism	N-formylphenylalanine	0,0002	0,0004	0,4661
Lipid	Secondary Bile Acid Metabolism	tauroursodeoxycholic acid sulfate (1)	0,0002	0,0004	0,4641
Lipid	Estrogenic Steroids	estrone 3-sulfate	0,0002	0,0005	0,4625
Amino Acid	Methionine, Cysteine, SAM and Taurine Metabolism	S-adenosylhomocysteine (SAH)	0,0002	0,0005	0,4618
Xenobiotics	Chemical	S-(3-hydroxypropyl)mercapturic acid (HPMA)	0,0002	0,0005	0,4611
Amino Acid	Urea cycle; Arginine and Proline Metabolism	N,N,N-trimethyl-alanylproline betaine (TMAP)	0,0002	0,0005	0,4598
Amino Acid	Alanine and Aspartate Metabolism	asparagine	0,0002	0,0005	0,4581
Lipid	Primary Bile Acid Metabolism	tauro-beta-muricholate	0,0002	0,0005	0,4577
Lipid	Fatty Acid Metabolism (Acyl Carnitine, Monounsaturated)	3-decenoylcarnitine	0,0002	0,0005	0,4571
Xenobiotics	Chemical	2-naphthol sulfate	0,0002	0,0005	0,4566
Cofactors and Vitamins	Nicotinate and Nicotinamide Metabolism	N1-methyl-2-pyridone-5-carboxamide	0,0002	0,0005	0,4563
Amino Acid	Leucine, Isoleucine and Valine Metabolism	N-acetylleucine	0,0003	0,0006	0,4546
Nucleotide	Pyrimidine Metabolism, Uracil containing	3-ureidopropionate	0,0003	0,0006	0,4536
Energy	TCA Cycle	malate	0,0003	0,0006	0,4532
Xenobiotics	Benzoate Metabolism	4-ethylphenylsulfate	0,0003	0,0006	0,4518
Xenobiotics	Drug - Analgesics, Anesthetics	N-ethylglycinexylidide	0,0003	0,0006	0,4515
Lipid	Primary Bile Acid Metabolism	glycocholate glucuronide (1)	0,0003	0,0007	0,4492
Lipid	Fatty Acid Metabolism (also BCAA Metabolism)	propionylcarnitine (C3)	0,0003	0,0007	0,4479
Lipid	Pregnenolone Steroids	21-hydroxypregnenolone monosulfate (1)	0,0004	0,0008	0,4456
Lipid	Long Chain Polyunsaturated Fatty Acid (n3 and n6)	arachidonate (20:4n6)	0,0004	0,0008	0,4446
Amino Acid	Lysine Metabolism	N2-acetyl,N6-methyllysine	0,0004	0,0008	0,4434
Amino Acid	Urea cycle; Arginine and Proline Metabolism	N2,N5-diacetylornithine	0,0004	0,0008	0,4430
Amino Acid	Urea cycle; Arginine and Proline Metabolism	N-acetylarginine	0,0004	0,0008	0,4426
Peptide	Gamma-glutamyl Amino Acid	gamma-glutamylphenylalanine	0,0004	0,0008	0,4424

Amino Acid	Glutamate Metabolism	beta-citrylglutamate	0,0004	0,0008	0,4421
Amino Acid	Tryptophan Metabolism	6-bromotryptophan	0,0004	0,0008	0,4421
Lipid	Fatty Acid, Dicarboxylate	2-hydroxysebacate	0,0004	0,0009	0,4392
Peptide	Modified Peptides	N,N-dimethyl-pro-pro	0,0005	0,0009	0,4377
Lipid	Pregnenolone Steroids	17alpha-hydroxypregnenolone 3-sulfate	0,0005	0,0009	0,4376
Nucleotide	Pyrimidine Metabolism, Cytidine containing	cytidine	0,0006	0,0012	0,4293
Lipid	Pregnenolone Steroids	17alpha-hydroxypregnanolone glucuronide	0,0006	0,0012	0,4290
Lipid	Fatty Acid, Dicarboxylate	glutarate (C5-DC)	0,0006	0,0013	0,4281
Lipid	Fatty Acid, Dicarboxylate	2-hydroxyadipate	0,0007	0,0013	0,4275
Amino Acid	Alanine and Aspartate Metabolism	hydroxyasparagine**	0,0007	0,0013	0,4271
Amino Acid	Tyrosine Metabolism	5-hydroxymethyl-2-furoic acid	0,0007	0,0013	0,4269
Amino Acid	Leucine, Isoleucine and Valine Metabolism	alpha-hydroxyisocaproate	0,0007	0,0013	0,4261
Amino Acid	Methionine, Cysteine, SAM and Taurine Metabolism	cysteine	0,0007	0,0013	0,4260
Lipid	Fatty Acid Metabolism (also BCAA Metabolism)	butyrylcarnitine (C4)	0,0007	0,0013	0,4259
Lipid	Secondary Bile Acid Metabolism	tauroursodeoxycholate	0,0007	0,0013	0,4257
Lipid	Fatty Acid Metabolism (Acyl Carnitine, Hydroxy)	3-hydroxyoctanoylcarnitine (2)	0,0007	0,0013	0,4249
Peptide	Dipeptide	valylleucine	0,0007	0,0014	0,4243
Amino Acid	Histidine Metabolism	histidine	0,0007	0,0014	0,4238
Carbohydrate	Pentose Metabolism	ribitol	0,0007	0,0014	0,4236
Partially Characterized Molecules	Partially Characterized Molecules	bilirubin degradation product, C16H18N2O5 (3)**	0,0008	0,0014	0,4230
Lipid	Sphingolipid Synthesis	sphinganine	0,0008	0,0015	0,4214
Energy	Oxidative Phosphorylation	phosphate	0,0009	0,0016	0,4187
Xenobiotics	Benzoate Metabolism	4-vinylphenol sulfate	0,0009	0,0016	0,4179
Amino Acid	Urea cycle; Arginine and Proline Metabolism	trans-4-hydroxyproline	0,0009	0,0017	0,4173
Partially Characterized Molecules	Partially Characterized Molecules	GlcNAc sulfate conjugate of C21H34O2 steroid**	0,0009	0,0017	0,4168
Amino Acid	Leucine, Isoleucine and Valine Metabolism	beta-hydroxyisovalerate	0,0010	0,0018	0,4147
Xenobiotics	Food Component/Plant	(2,4 or 2,5)-dimethylphenol sulfate	0,0010	0,0018	0,4137
Lipid	Fatty Acid Metabolism (Acyl Carnitine, Dicarboxylate)	octadecenedioylcarnitine (C18:1-DC)*	0,0011	0,0019	0,4120
Cofactors and Vitamins	Vitamin B6 Metabolism	pyridoxate	0,0011	0,0019	0,4119

Lipid	Carnitine Metabolism	carnitine	0,0011	0,0020	0,4100
Amino Acid	Glutathione Metabolism	cysteinylglycine	0,0012	0,0021	0,4093
Lipid	Fatty Acid Metabolism (Acyl Carnitine, Short Chain)	acetylcarnitine (C2)	0,0012	0,0021	0,4083
Amino Acid	Guanidino and Acetamido Metabolism	4-guanidinobutanoate	0,0012	0,0021	0,4076
Xenobiotics	Chemical	3-acetylphenol sulfate	0,0013	0,0022	0,4067
Lipid	Fatty Acid Metabolism (Acyl Choline)	arachidonoylcholine	0,0013	0,0022	0,4064
Peptide	Acetylated Peptides	4-hydroxyphenylacetylglutamine	0,0013	0,0022	0,4056
Peptide	Gamma-glutamyl Amino Acid	gamma-glutamylthreonine	0,0014	0,0024	0,4038
Carbohydrate	Pentose Metabolism	lyxonate	0,0014	0,0024	0,4035
Lipid	Fatty Acid Metabolism (Acyl Carnitine, Hydroxy)	3-hydroxyhexanoylcarnitine (1)	0,0014	0,0024	0,4029
Energy	TCA Cycle	fumarate	0,0014	0,0024	0,4025
Lipid	Fatty Acid Metabolism (Acyl Carnitine, Medium Chain)	octanoylcarnitine (C8)	0,0015	0,0025	0,4016
Amino Acid	Methionine, Cysteine, SAM and Taurine Metabolism	cysteine sulfinic acid	0,0015	0,0025	0,4014
Xenobiotics	Food Component/Plant	2,8-quinolinediol sulfate	0,0015	0,0025	0,4011
Amino Acid	Lysine Metabolism	N2-acetyl,N6,N6-dimethyllysine	0,0015	0,0025	0,4009
Amino Acid	Lysine Metabolism	N,N,N-trimethyl-5-aminovalerate	0,0016	0,0026	0,3989
Lipid	Corticosteroids	cortolone glucuronide (1)	0,0016	0,0027	0,3984
Energy	TCA Cycle	succinate	0,0017	0,0027	0,3975
Amino Acid	Alanine and Aspartate Metabolism	N-carbamoylalanine	0,0017	0,0028	0,3965
Energy	TCA Cycle	alpha-ketoglutarate	0,0017	0,0028	0,3959
Lipid	Androgenic Steroids	androstenediol (3alpha, 17alpha) monosulfate (3)	0,0018	0,0028	0,3957
Lipid	Fatty Acid Metabolism (Acyl Carnitine, Dicarboxylate)	pimeloylcarnitine/3-methyladipoylcarnitine (C7-DC)	0,0018	0,0028	0,3956
Amino Acid	Glutamate Metabolism	alpha-ketoglutaramate*	0,0018	0,0029	0,3952
Lipid	Fatty Acid Metabolism (Acyl Choline)	docosahexaenoylcholine	0,0018	0,0029	0,3945
Lipid	Androgenic Steroids	androsterone glucuronide	0,0019	0,0030	0,3934
Lipid	Corticosteroids	tetrahydrocortisone glucuronide (5)	0,0019	0,0030	0,3933
Lipid	Fatty Acid Metabolism (Acyl Choline)	dihomo-linolenoyl-choline	0,0019	0,0030	0,3932
Xenobiotics	Food Component/Plant	mannonate*	0,0020	0,0032	0,3907
Amino Acid	Methionine, Cysteine, SAM and Taurine	lanthionine	0,0021	0,0032	0,3903

	Metabolism				
Lipid	Phospholipid Metabolism	glycerophosphoserine*	0,0021	0,0033	0,3898
Cofactors and Vitamins	Ascorbate and Aldarate Metabolism	gulonate*	0,0021	0,0033	0,3895
Lipid	Androgenic Steroids	5alpha-androstan-3alpha,17beta-diol disulfate	0,0022	0,0034	0,3883
Amino Acid	Lysine Metabolism	N-acetyl-cadaverine	0,0023	0,0035	0,3869
Amino Acid	Phenylalanine Metabolism	2-hydroxyphenylacetate	0,0023	0,0035	0,3868
Amino Acid	Urea cycle; Arginine and Proline Metabolism	arginine	0,0023	0,0035	0,3868
Lipid	Phosphatidylserine (PS)	1-stearoyl-2-oleoyl-GPS (18:0/18:1)	0,0024	0,0037	0,3842
Lipid	Corticosteroids	tetrahydrocortisol glucuronide	0,0025	0,0038	0,3838
Xenobiotics	Chemical	O-sulfo-tyrosine	0,0025	0,0038	0,3832
Partially Characterized Molecules	Partially Characterized Molecules	N-acetylglucosamine conjugate of C24H40O4 bile acid**	0,0025	0,0039	0,3827
Lipid	Fatty Acid Metabolism (also BCAA Metabolism)	propionylglycine	0,0026	0,0039	0,3825
Cofactors and Vitamins	Pantothenate and CoA Metabolism	pantoate	0,0026	0,0039	0,3819
Lipid	Fatty Acid Metabolism (Acyl Carnitine, Dicarboxylate)	octadecanedioylcarnitine (C18-DC)*	0,0027	0,0041	0,3806
Xenobiotics	Drug - Psychoactive	THC carboxylic acid	0,0028	0,0042	0,3793
Xenobiotics	Benzoate Metabolism	methyl-4-hydroxybenzoate sulfate	0,0028	0,0042	0,3792
Amino Acid	Leucine, Isoleucine and Valine Metabolism	ethylmalonate	0,0029	0,0043	0,3784
Lipid	Secondary Bile Acid Metabolism	glycoursodeoxycholate	0,0029	0,0043	0,3779
Nucleotide	Purine Metabolism, Guanine containing	guanosine	0,0031	0,0045	0,3761
Xenobiotics	Drug - Psychoactive	THC carboxylic acid glucuronide	0,0031	0,0046	0,3756
Xenobiotics	Food Component/Plant	2-aminophenol sulfate	0,0032	0,0046	0,3748
Amino Acid	Glycine, Serine and Threonine Metabolism	dimethylglycine	0,0032	0,0047	0,3745
Lipid	Fatty Acid Metabolism (Acyl Choline)	stearoylcholine*	0,0033	0,0047	0,3738
Lipid	Fatty Acid, Dicarboxylate	tetradecanedioate (C14-DC)	0,0034	0,0050	0,3720
Amino Acid	Histidine Metabolism	hydantoin-5-propionate	0,0036	0,0052	0,3697
Lipid	Sphingosines	sphingosine	0,0036	0,0052	0,3697
Lipid	Androgenic Steroids	androstenediol (3alpha, 17alpha) monosulfate (2)	0,0037	0,0053	0,3694
Peptide	Gamma-glutamyl Amino Acid	gamma-glutamylmethionine	0,0038	0,0055	0,3679
Lipid	Fatty Acid Metabolism (Acyl Choline)	palmitoylcholine	0,0039	0,0056	0,3669

Lipid	Endocannabinoid	N-palmitoylserine	0,0041	0,0058	0,3651
Peptide	Fibrinogen Cleavage Peptide	fibrinopeptide A (2-15)**	0,0042	0,0059	0,3643
Amino Acid	Urea cycle; Arginine and Proline Metabolism	proline	0,0042	0,0060	0,3641
Peptide	Fibrinogen Cleavage Peptide	fibrinopeptide A (5-16)*	0,0043	0,0061	0,3633
Lipid	Fatty Acid Metabolism (Acyl Carnitine, Dicarboxylate)	suberoylcarnitine (C8-DC)	0,0044	0,0061	0,3631
Peptide	Gamma-glutamyl Amino Acid	gamma-glutamylglutamate	0,0044	0,0061	0,3627
Lipid	Eicosanoid	leukotriene B5	0,0044	0,0061	0,3625
Carbohydrate	Pentose Metabolism	arabonate/xylonate	0,0045	0,0062	0,3623
Amino Acid	Leucine, Isoleucine and Valine Metabolism	methylsuccinoylcarnitine	0,0047	0,0065	0,3602
Xenobiotics	Food Component/Plant	tartronate (hydroxymalonate)	0,0047	0,0065	0,3598
Amino Acid	Lysine Metabolism	pipecolate	0,0054	0,0074	0,3546
Xenobiotics	Drug - Analgesics, Anesthetics	2-hydroxyibuprofen	0,0054	0,0074	0,3546
Lipid	Androgenic Steroids	16alpha-hydroxy DHEA 3-sulfate	0,0056	0,0076	0,3536
Carbohydrate	Glycolysis, Gluconeogenesis, and Pyruvate Metabolism	lactate	0,0056	0,0076	0,3532
Amino Acid	Glutathione Metabolism	2-hydroxybutyrate/2-hydroxyisobutyrate	0,0057	0,0077	0,3525
Lipid	Fatty Acid Metabolism (Acyl Carnitine, Long Chain Saturated)	stearoylcarnitine (C18)	0,0058	0,0078	0,3519
Lipid	Phospholipid Metabolism	glycerophosphoethanolamine	0,0059	0,0079	0,3515
Lipid	Corticosteroids	cortisol 21-sulfate	0,0061	0,0082	0,3501
Lipid	Fatty Acid, Dihydroxy	9,10-DiHOME	0,0063	0,0084	0,3490
Xenobiotics	Benzoate Metabolism	4-hydroxyhippurate	0,0065	0,0086	0,3478
Xenobiotics	Benzoate Metabolism	3-ethylphenylsulfate	0,0065	0,0086	0,3475
Lipid	Secondary Bile Acid Metabolism	taurolithocholate	0,0067	0,0089	0,3463
Peptide	Gamma-glutamyl Amino Acid	gamma-glutamyl-2-aminobutyrate	0,0068	0,0089	0,3460
Peptide	Gamma-glutamyl Amino Acid	gamma-glutamyltyrosine	0,0071	0,0093	0,3440
Xenobiotics	Drug - Analgesics, Anesthetics	carboxyibuprofen	0,0071	0,0093	0,3439
Cofactors and Vitamins	Tocopherol Metabolism	alpha-tocopherol	0,0071	0,0093	0,3438
Amino Acid	Phenylalanine Metabolism	4-hydroxyphenylacetate	0,0072	0,0094	0,3435
Lipid	Fatty Acid Metabolism (Acyl Glutamine)	hexanoylglutamine	0,0072	0,0094	0,3434
Amino Acid	Phenylalanine Metabolism	phenylalanine	0,0073	0,0094	0,3431

Amino Acid	Urea cycle; Arginine and Proline Metabolism	dimethylarginine (SDMA + ADMA)	0,0073	0,0094	0,3428
Xenobiotics	Drug - Topical Agents	2,6-dihydroxybenzoic acid	0,0074	0,0095	0,3426
Lipid	Fatty Acid Metabolism (Acyl Choline)	linoleoylcholine*	0,0074	0,0095	0,3422
Nucleotide	Purine Metabolism, (Hypo)Xanthine/Inosine containing	urate	0,0075	0,0096	0,3417
Amino Acid	Tryptophan Metabolism	7-hydroxyindole sulfate	0,0077	0,0098	0,3408
Amino Acid	Lysine Metabolism	N,N-dimethyl-5-aminovalerate	0,0080	0,0101	0,3391
Amino Acid	Urea cycle; Arginine and Proline Metabolism	argininosuccinate	0,0080	0,0101	0,3391
Lipid	Secondary Bile Acid Metabolism	taurodeoxycholate	0,0080	0,0101	0,3391
Amino Acid	Methionine, Cysteine, SAM and Taurine Metabolism	alpha-ketobutyrate	0,0086	0,0108	0,3363
Cofactors and Vitamins	Tocopherol Metabolism	alpha-CEHC	0,0087	0,0109	0,3359
Lipid	Lysophospholipid	1-palmitoyl-GPG (16:0)*	0,0089	0,0111	0,3349
Amino Acid	Methionine, Cysteine, SAM and Taurine Metabolism	methionine	0,0090	0,0112	0,3344
Lipid	Fatty Acid, Dicarboxylate	hexadecanedioate (C16-DC)	0,0092	0,0113	0,3337
Amino Acid	Glutathione Metabolism	cys-gly, oxidized	0,0092	0,0113	0,3336
Nucleotide	Pyrimidine Metabolism, Cytidine containing	2'-O-methylcytidine	0,0092	0,0113	0,3336
Lipid	Fatty Acid Metabolism (Acyl Carnitine, Monounsaturated)	ximenoylcarnitine (C26:1)*	0,0092	0,0113	0,3335
Nucleotide	Purine Metabolism, (Hypo)Xanthine/Inosine containing	xanthosine	0,0094	0,0115	0,3326
Xenobiotics	Drug - Analgesics, Anesthetics	ibuprofen acyl glucuronide	0,0094	0,0115	0,3326
Lipid	Phosphatidylserine (PS)	1-stearoyl-2-arachidonoyl-GPS (18:0/20:4)	0,0095	0,0116	0,3322
Xenobiotics	Drug - Antibiotic	meropenem	0,0096	0,0117	0,3317
Carbohydrate	Disaccharides and Oligosaccharides	sucrose	0,0098	0,0119	0,3308
Lipid	Fatty Acid Metabolism (Acyl Choline)	eicosapentaenoylcholine	0,0101	0,0122	0,3296
Lipid	Androgenic Steroids	etiocholanolone glucuronide	0,0102	0,0123	0,3291
Lipid	Sterol	7alpha-hydroxy-3-oxo-4-cholestenoate (7-Hoca)	0,0106	0,0127	0,3276
Carbohydrate	Glycogen Metabolism	maltose	0,0107	0,0128	0,3272
Amino Acid	Alanine and Aspartate Metabolism	aspartate	0,0108	0,0128	0,3270
Xenobiotics	Benzoate Metabolism	guaiacol sulfate	0,0108	0,0128	0,3269
Peptide	Dipeptide	isoleucylglycine	0,0109	0,0128	0,3266

Lipid	Medium Chain Fatty Acid	pelargonate (9:0)	0,0109	0,0128	0,3266
Lipid	Fatty Acid, Dicarboxylate	tridecenedioate (C13:1-DC)*	0,0109	0,0129	0,3263
Peptide	Fibrinogen Cleavage Peptide	fibrinopeptide A (3-15)**	0,0111	0,0131	0,3256
Xenobiotics	Drug - Analgesics, Anesthetics	lidocaine	0,0117	0,0137	0,3235
Peptide	Gamma-glutamyl Amino Acid	gamma-glutamylisoleucine*	0,0119	0,0139	0,3226
Cofactors and Vitamins	Tocopherol Metabolism	gamma-tocopherol/beta-tocopherol	0,0121	0,0141	0,3219
Amino Acid	Leucine, Isoleucine and Valine Metabolism	N-acetylisoleucine	0,0122	0,0141	0,3217
Peptide	Gamma-glutamyl Amino Acid	gamma-glutamyl-alpha-lysine	0,0122	0,0141	0,3217
Lipid	Androgenic Steroids	andro steroid monosulfate C19H28O6S (1)*	0,0123	0,0141	0,3214
Amino Acid	Glutamate Metabolism	glutamate	0,0123	0,0142	0,3212
Cofactors and Vitamins	Ascorbate and Aldarate Metabolism	ascorbic acid 2-sulfate	0,0126	0,0145	0,3202
Peptide	Fibrinogen Cleavage Peptide	fibrinopeptide A (7-16)*	0,0127	0,0145	0,3201
Cofactors and Vitamins	Nicotinate and Nicotinamide Metabolism	N1-methyl-4-pyridone-3-carboxamide	0,0128	0,0146	0,3196
Lipid	Androgenic Steroids	5alpha-androstan-3alpha,17beta-diol 17-glucuronide	0,0131	0,0149	0,3186
Lipid	Endocannabinoid	N-stearoylserine*	0,0131	0,0149	0,3185
Peptide	Fibrinogen Cleavage Peptide	fibrinopeptide B (1-13)**	0,0139	0,0157	0,3161
Lipid	Fatty Acid, Dicarboxylate	heptenedioate (C7:1-DC)*	0,0143	0,0161	0,3147
Lipid	Lysophospholipid	1-linoleoyl-GPG (18:2)*	0,0144	0,0161	0,3146
Amino Acid	Tryptophan Metabolism	N-acetyltryptophan	0,0148	0,0166	0,3131
Carbohydrate	Pentose Metabolism	arabinose	0,0154	0,0171	0,3116
Lipid	Fatty Acid, Monohydroxy	2-hydroxyoctanoate	0,0154	0,0171	0,3116
Lipid	Short Chain Fatty Acid	butyrate/isobutyrate (4:0)	0,0157	0,0175	0,3105
Xenobiotics	Benzoate Metabolism	4-acetylphenol sulfate	0,0160	0,0177	0,3099
Peptide	Fibrinogen Cleavage Peptide	fibrinopeptide A (4-15)**	0,0162	0,0179	0,3092
Lipid	Medium Chain Fatty Acid	caproate (6:0)	0,0165	0,0182	0,3084
Peptide	Fibrinogen Cleavage Peptide	fibrinopeptide A (8-16)**	0,0168	0,0185	0,3076
Xenobiotics	Drug - Analgesics, Anesthetics	ibuprofen	0,0169	0,0185	0,3074
Lipid	Primary Bile Acid Metabolism	glyco-beta-muricholate**	0,0176	0,0192	0,3056
Amino Acid	Leucine, Isoleucine and Valine Metabolism	2-hydroxy-3-methylvalerate	0,0178	0,0194	0,3049

Amino Acid	Tyrosine Metabolism	homovanillate (HVA)	0,0181	0,0196	0,3042
Lipid	Long Chain Polyunsaturated Fatty Acid (n3 and n6)	dihomo-linolenate (20:3n3 or n6)	0,0181	0,0196	0,3042
Lipid	Fatty Acid, Monohydroxy	5-hydroxyhexanoate	0,0182	0,0196	0,3041
Amino Acid	Lysine Metabolism	N6,N6-dimethyllysine	0,0187	0,0201	0,3029
Amino Acid	Leucine, Isoleucine and Valine Metabolism	2-methylbutyrylcarnitine (C5)	0,0192	0,0206	0,3017
Amino Acid	Leucine, Isoleucine and Valine Metabolism	leucine	0,0193	0,0207	0,3014
Lipid	Corticosteroids	cortisone	0,0194	0,0207	0,3011
Xenobiotics	Chemical	diglycerol	0,0195	0,0208	0,3010
Carbohydrate	Glycolysis, Gluconeogenesis, and Pyruvate Metabolism	1,5-anhydroglucitol (1,5-AG)	0,0195	0,0208	0,3008
Nucleotide	Purine Metabolism, (Hypo)Xanthine/Inosine containing	inosine	0,0196	0,0209	0,3005
Amino Acid	Glycine, Serine and Threonine Metabolism	glycine	0,0199	0,0210	0,3000
Amino Acid	Leucine, Isoleucine and Valine Metabolism	3-methylglutarylcarnitine (2)	0,0202	0,0213	0,2992
Xenobiotics	Tobacco Metabolite	2-hydroxyfluorene sulfate	0,0207	0,0217	0,2982
Xenobiotics	Chemical	benzoylcarnitine*	0,0208	0,0219	0,2979
Xenobiotics	Drug - Analgesics, Anesthetics	3-(N-acetyl-cystein-S-yl) acetaminophen	0,0211	0,0221	0,2973
Cofactors and Vitamins	Vitamin A Metabolism	retinal	0,0212	0,0222	0,2969
Peptide	Dipeptide	threonylphenylalanine	0,0219	0,0228	0,2955
Carbohydrate	Pentose Metabolism	ribonate	0,0222	0,0230	0,2949
Peptide	Acetylated Peptides	phenylacetylglycine	0,0224	0,0232	0,2945
Carbohydrate	Glycolysis, Gluconeogenesis, and Pyruvate Metabolism	pyruvate	0,0224	0,0232	0,2944
Amino Acid	Leucine, Isoleucine and Valine Metabolism	3-hydroxy-2-ethylpropionate	0,0225	0,0232	0,2943
Amino Acid	Glutamate Metabolism	S-1-pyrroline-5-carboxylate	0,0227	0,0234	0,2938
Lipid	Eicosanoid	12-HHTrE	0,0240	0,0246	0,2912
Peptide	Dipeptide	leucylalanine	0,0242	0,0247	0,2908
Xenobiotics	Chemical	succinimide	0,0242	0,0247	0,2908
Nucleotide	Pyrimidine Metabolism, Cytidine containing	cytosine	0,0245	0,0249	0,2902
Cofactors and Vitamins	Vitamin A Metabolism	carotene diol (2)	0,0252	0,0256	0,2888
Amino Acid	Tryptophan Metabolism	tryptophan	0,0254	0,0257	0,2885
Amino Acid	Tryptophan Metabolism	serotonin	0,0255	0,0257	0,2884

Lipid	Medium Chain Fatty Acid	cis-4-decenoate (10:1n6)*	0,0263	0,0264	0,2869
Lipid	Fatty Acid Metabolism (Acyl Carnitine, Monounsaturated)	myristoleoylcarnitine (C14:1)*	0,0262	0,0264	0,2869
Peptide	Acetylated Peptides	phenylacetylglutamine	0,0270	0,0271	0,2856
Xenobiotics	Drug - Analgesics, Anesthetics	morphine-6-glucuronide	0,0272	0,0273	0,2851
Xenobiotics	Drug - Analgesics, Anesthetics	morphine	0,0277	0,0277	0,2842
Nucleotide	Pyrimidine Metabolism, Uracil containing	uridine	0,0278	0,0277	0,2841
Partially Characterized Molecules	Partially Characterized Molecules	glycine conjugate of C10H12O2*	0,0284	0,0282	0,2830
Nucleotide	Purine Metabolism, Guanine containing	guanine	0,0290	0,0287	0,2821
Xenobiotics	Drug - Analgesics, Anesthetics	morphine-3-glucuronide	0,0292	0,0289	0,2817
Lipid	Fatty Acid, Dicarboxylate	octadecadienedioate (C18:2-DC)*	0,0297	0,0293	0,2809
Lipid	Fatty Acid, Dicarboxylate	adipate (C6-DC)	0,0298	0,0293	0,2807
Lipid	Lysophospholipid	1-arachidonoyl-GPA (20:4)	0,0302	0,0296	0,2801
Xenobiotics	Drug - Antibiotic	clindamycin	0,0304	0,0297	0,2798
Amino Acid	Urea cycle; Arginine and Proline Metabolism	homocitrulline	0,0308	0,0300	0,2792
Lipid	Androgenic Steroids	androsterone sulfate	0,0308	0,0300	0,2791
Xenobiotics	Chemical	methylnaphthyl sulfate (2)*	0,0309	0,0300	0,2789
Amino Acid	Leucine, Isoleucine and Valine Metabolism	3-hydroxyisobutyrate	0,0316	0,0307	0,2778
Partially Characterized Molecules	Partially Characterized Molecules	bilirubin degradation product, C16H18N2O5 (1)**	0,0317	0,0307	0,2777
Lipid	Androgenic Steroids	5alpha-androstan-3alpha,17beta-diol monosulfate (1)	0,0325	0,0313	0,2764
Peptide	Gamma-glutamyl Amino Acid	gamma-glutamylglycine	0,0326	0,0313	0,2763
Lipid	Fatty Acid Synthesis	malonate	0,0331	0,0318	0,2755
Lipid	Fatty Acid, Monohydroxy	3-hydroxystearate	0,0340	0,0325	0,2743
Xenobiotics	Chemical	2,4-di-tert-butylphenol	0,0341	0,0325	0,2741
Amino Acid	Methionine, Cysteine, SAM and Taurine Metabolism	S-methylcysteine sulfoxide	0,0356	0,0339	0,2719
Lipid	Sphingolipid Synthesis	sphingadienine	0,0356	0,0339	0,2718
Lipid	Androgenic Steroids	androstenediol (3beta,17beta) disulfate (1)	0,0360	0,0341	0,2714
Lipid	Fatty Acid, Dicarboxylate	octadecenedioate (C18:1-DC)	0,0362	0,0342	0,2711
Xenobiotics	Drug - Antibiotic	penicillin G	0,0365	0,0344	0,2706

Lipid	Androgenic Steroids	5alpha-androstan-3alpha,17alpha-diol monosulfate	0,0366	0,0344	0,2705
Xenobiotics	Drug - Gastrointestinal	pantoprazole	0,0367	0,0345	0,2703
Carbohydrate	Fructose, Mannose and Galactose Metabolism	fructose	0,0374	0,0350	0,2694
Cofactors and Vitamins	Vitamin B6 Metabolism	pyridoxal	0,0376	0,0352	0,2691
Lipid	Glycerolipid Metabolism	glycerol 3-phosphate	0,0377	0,0352	0,2689
Xenobiotics	Chemical	perfluorohexanesulfonate (PFHxS)	0,0378	0,0352	0,2688
Xenobiotics	Food Component/Plant	eugenol sulfate	0,0382	0,0355	0,2682
Nucleotide	Purine Metabolism, (Hypo)Xanthine/Inosine containing	allantoin	0,0383	0,0355	0,2681
Cofactors and Vitamins	Ascorbate and Aldarate Metabolism	oxalate (ethanedioate)	0,0395	0,0366	0,2665
Xenobiotics	Food Component/Plant	dihydrocaffeate sulfate (2)	0,0398	0,0367	0,2662
Xenobiotics	Food Component/Plant	2-piperidinone	0,0400	0,0367	0,2660
Lipid	Sterol	3beta,7alpha-dihydroxy-5-cholestenoate	0,0400	0,0367	0,2659
Peptide	Fibrinogen Cleavage Peptide	fibrinopeptide A, des-ala(1)*	0,0411	0,0377	0,2645
Peptide	Fibrinogen Cleavage Peptide	fibrinopeptide B (1-9)**	0,0413	0,0378	0,2643
Lipid	Fatty Acid, Dicarboxylate	sebacate (C10-DC)	0,0423	0,0386	0,2631
Lipid	Fatty Acid Metabolism (Acyl Carnitine, Polyunsaturated)	linolenoylcarnitine (C18:3)*	0,0433	0,0394	0,2618
Lipid	Secondary Bile Acid Metabolism	lithocholate sulfate (1)	0,0435	0,0395	0,2615
Xenobiotics	Xanthine Metabolism	1-methylurate	0,0437	0,0396	0,2614
Cofactors and Vitamins	Vitamin A Metabolism	retinol (vitamin A)	0,0459	0,0415	0,2588
Lipid	Fatty Acid Metabolism (Acyl Carnitine, Long Chain Saturated)	lignoceroylcarnitine (C24)*	0,0466	0,0420	0,2580
Amino Acid	Tryptophan Metabolism	N-formylanthranilic acid	0,0472	0,0425	0,2573
Lipid	Fatty Acid, Monohydroxy	2-hydroxystearate	0,0474	0,0426	0,2570
Xenobiotics	Food Component/Plant	Pyrraline	0,0478	0,0429	0,2566
Peptide	Dipeptide	Phenylalanylalanine	0,0496	0,0444	0,2546

Table S9. Correlation between individual the 56 steroid hormone metabolites and the maximal total SOFA score. The table presents the classification and identity of the metabolite together with the corresponding p-value and correlation value from the correlation analysis and the percentage of Sepsis-3 and Sepsis-2 patients with detectable levels. A correlation coefficient of 0.4-0.5 is indicated by gery shadow, a valie of 0.5-0.6 by dark grey shadow and a value >0.06 by dark grey shadow and the value in bold. P-values between 0.05-0.01 are also marked with gery shadow and values below 0.01 with dark grey shadow (* The metabolite identity not confirmed based on a standard).

**Biochemical Subpathway/Metabolite Name**	**p-value**	**Correlation**	**% detectable**	
			**Sepsis-3**	**Sepsis-2**
**Sterols (7 metabolites)**				
Cholesterol	0,5103	0,0867	100%	100%
cholesterol sulfate	0,2975	0,1367	100	100
7alpha-hydroxy-3-oxo-4-cholestenoate (7-Hoca)	0,0106	0,3276	100	100
3beta,7alpha-dihydroxy-5-cholestenoate	0,0400	0,2659	100	100
3beta-hydroxy-5-cholestenoate	0,2116	0,1636	91	76
4-cholesten-3-one	0,6933	0,0520	37	28
Campesterol	0,1793	0,1757	100	100
**Pregnenolone steroids (9 metabolites)**				
pregnenolone sulfate	0,0000	0,5643	66	60
17alpha-hydroxypregnenolone 3-sulfate	0,0005	0,4376	100	96
17alpha-hydroxypregnanolone glucuronide	0,0006	0,4290	69	56
21-hydroxypregnenolone monosulfate (1)	0,0004	0,4456	100	100
21-hydroxypregnenolone disulfate	0,0000	**0,6244**	100	100
pregnenediol sulfate (C21H34O5S)*	0,0000	0,5592	100	100
pregnenediol disulfate (C21H34O8S2)*	0,0000	**0,6248**	100	100
pregnenetriol sulfate*	0,0000	0,5409	100	100
pregnenetriol disulfate*	0,0000	0,5848	54	44
**Progestin steroids (7 metabolites)**				
5alpha-pregnan-3beta-ol,20-one sulfate	0,0000	**0,6636**	100	96
5alpha-pregnan-3beta,20beta-diol monosulfate (1)	0,0000	**0,6905**	97	96
5alpha-pregnan-3beta,20alpha-diol monosulfate (2)	0,0000	0,5298	100	100
5alpha-pregnan-3beta,20alpha-diol disulfate	0,0001	0,4870	91	80
5alpha-pregnan-diol disulfate	0,0000	**0,6320**	97	100
pregnanediol-3-glucuronide	0,0001	0,4839	80	52
pregnanolone/allopregnanolone sulfate	0,0000	**0,6579**	71	64
**Corticosteroids (8 metabolites)**				
Corticosterone	0,2252	0,1589	100	100
Cortisol	0,4517	0,0990	89	56
cortisol 21-sulfate	0,0061	0,3501	100	100
tetrahydrocortisol glucuronide	0,0025	0,3838	94	80
tetrahydrocortisol sulfate (1)	0,0001	0,4908	100	100
Cortisone	0,0194	0,3011	100	92
tetrahydrocortisone glucuronide (5)	0,0019	0,3933	100	100
cortolone glucuronide (1)	0,0016	0,3984	71	48

**Androgenic steroids (25 metabolites)**				
11-ketoetiocholanolone glucuronide	0,8753	0,0207	100	100
dehydroepiandrosterone sulfate (DHEA-S)	0,1720	0,1786	100	100
16alpha-hydroxy DHEA 3-sulfate	0,0056	0,3536	97	96
androsterone glucuronide	0,0019	0,3934	100	100
epiandrosterone sulfate	0,2045	0,1662	100	100
androsterone sulfate	0,0308	0,2791	97	100
etiocholanolone glucuronide	0,0102	0,3291	91	92
11beta-hydroxyetiocholanolone glucuronide*	0,6600	-0,0580	71	56
5alpha-androstan-3alpha,17alpha-diol monosulfate	0,0366	0,2705	77	48
5alpha-androstan-3alpha,17alpha-diol disulfate	0,0000	0,5751	100	100
androstenediol (3beta,17beta) monosulfate (1)	0,1363	0,1946	97	92
androstenediol (3beta,17beta) monosulfate (2)	0,5335	-0,0820	100	100
androstenediol (3beta,17beta) disulfate (1)	0,0360	0,2714	100	100
androstenediol (3beta,17beta) disulfate (2)	0,0000	0,5510	100	100
androstenediol (3alpha, 17alpha) monosulfate (2)	0,0037	0,3694	97	96
androstenediol (3alpha, 17alpha) monosulfate (3)	0,0018	0,3957	94	92
5alpha-androstan-3alpha,17beta-diol monosulfate (1)	0,0325	0,2764	71	72
5alpha-androstan-3alpha,17beta-diol monosulfate (2)	0,7326	-0,0450	86	72
5alpha-androstan-3alpha,17beta-diol disulfate	0,0022	0,3883	40	36
5alpha-androstan-3alpha,17beta-diol 17-glucuronide	0,0131	0,3186	86	80
5alpha-androstan-3beta,17beta-diol monosulfate (2)	0,1891	0,1719	100	100
5alpha-androstan-3beta,17beta-diol disulfate	0,0600	0,2442	91	76
5alpha-androstan-3beta,17alpha-diol disulfate	0,0962	0,2167	94	88
andro steroid monosulfate C19H28O6S (1)*	0,0123	0,3214	97	100
11beta-hydroxyandrosterone glucuronide	0,0532	0,2508	83	52
**Estrogenic steroids (1 metabolite)**				
estrone 3-sulfate	0,0002	0,4625	100	100

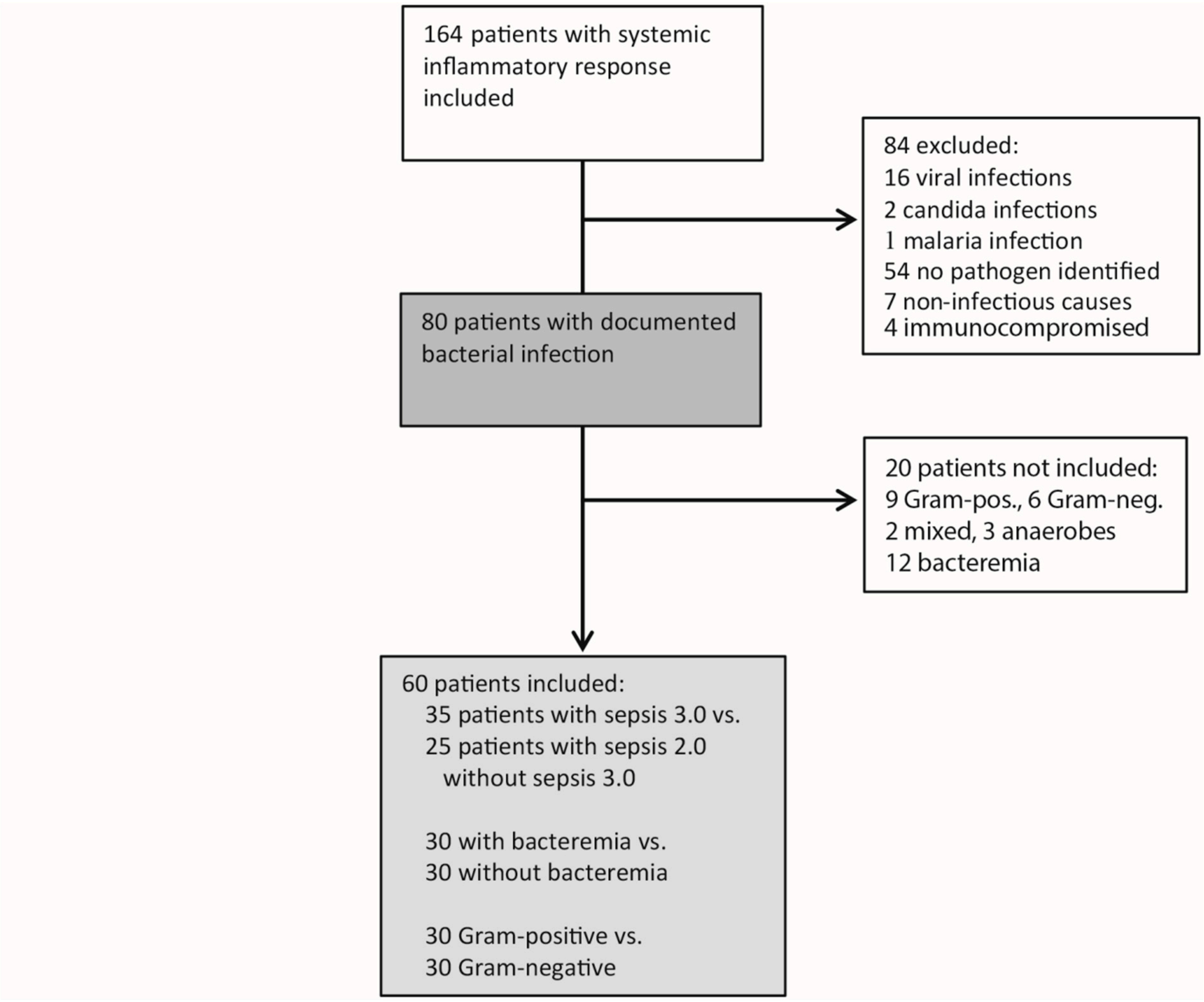

Figure S1. Selection of patients for the present study. Our study is based on a previous clinical study including 164 patients with a systemic inflammatory response; 84 patients were excluded (see upper right box) because they either were immunocompromized or had no documented bacterial infection. We then excluded 20 additional patients (lower right box) because they had mixed infections, infections with exceptional bacteria, or infection could not be documented by bacterial cutures (19 patients). One additional randomly selected patient with a Gram-negative infection was also excluded to have two groups with 30 patients each; our present study thereby included 60 patients. The 20 last excluded patients (see lower right box) included 12 patients with bacteremia, the main characteristics of the included patients are given in the lower grey box.

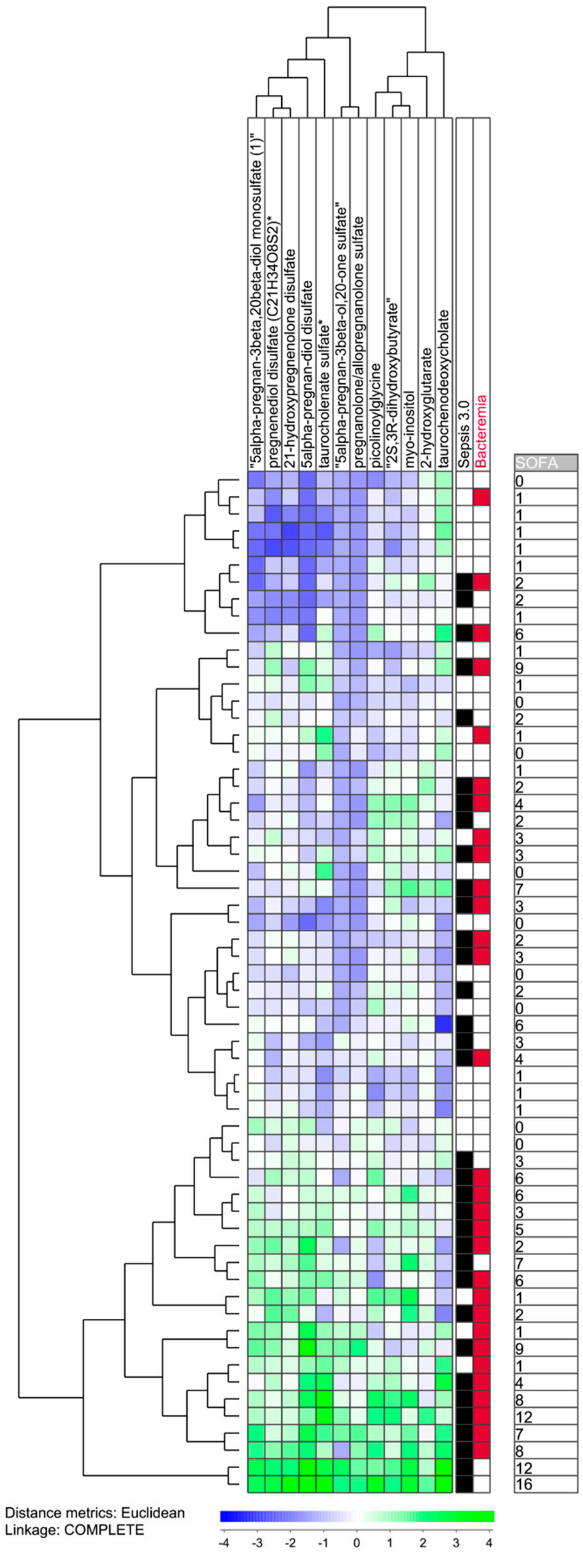

Figure S2. Subclassification of sepsis patients based on lipid metabolites that showed a strong correlation with the total SOFA score. The unsupervised hierarchical clustering analysis was based on 12 lipid metabolites showing a correlation with the Pearson's correlation test corresponding to a p-value <0.005 and a correlation factor >0.6. All metabolites showed detectable levels for at least 10 patients The characteristics of each individual patient (fulfilling Sepsis-3 criteria, detection of bacteremia, total SOFA score) are indicated to the right in the figure.

METHODOLOGICAL STRATEGIES USED BY METABOLON FOR ANALYSIS OF SERUM SAMPLES

The Metabolon platform

Sample Accessioning: Following receipt, samples were inventoried and immediately stored at -80°C. Each sample received was accessioned into the Metabolon LIMS system and was assigned by the LIMS a unique identifier that was associated with the original source identifier only. This identifier was used to track all sample handling, tasks, results, etc. The samples (and all derived aliquots) were tracked by the LIMS system. All portions of any sample were automatically assigned their own unique identifiers by the LIMS when a new task was created; the relationship of these samples was also tracked. All samples were maintained at -80°C until processed.

Sample Preparation: Samples were prepared using the automated MicroLab STAR® system from Hamilton Company. Several recovery standards were added prior to the first step in the extraction process for QC purposes. To remove protein, dissociate small molecules bound to protein or trapped in the precipitated protein matrix, and to recover chemically diverse metabolites, proteins were precipitated with methanol under vigorous shaking for 2 min (Glen Mills GenoGrinder 2000) followed by centrifugation. The resulting extract was divided into five fractions: two for analysis by two separate reverse phase (RP)/UPLC-MS/MS methods with positive ion mode electrospray ionization (ESI), one for analysis by RP/UPLC-MS/MS with negative ion mode ESI, one for analysis by HILIC/UPLC-MS/MS with negative ion mode ESI, and one sample was reserved for backup. Samples were placed briefly on a TurboVap® (Zymark) to remove the organic solvent. The sample extracts were stored overnight under nitrogen before preparation for analysis.

QA/QC: Several types of controls were analyzed in concert with the experimental samples: a pooled matrix sample generated by taking a small volume of each experimental sample (or alternatively, use of a pool of well-characterized human plasma) served as a technical replicate throughout the data set; extracted water samples served as process blanks; and a cocktail of QC standards that were carefully chosen not to interfere with the measurement of endogenous compounds were spiked into every analyzed sample, allowed instrument performance monitoring and aided chromatographic alignment. Tables 1 and 2 describe these QC samples and standards. Instrument variability was determined by calculating the median relative standard deviation (RSD) for the standards that were added to each sample prior to injection into the mass spectrometers. Overall process variability was determined by calculating the median RSD for all endogenous metabolites (i.e., non-instrument standards) present in 100% of the pooled matrix samples. Experimental samples were randomized across the platform run with QC samples spaced evenly among the injections, as outlined in Figure 1.

Table S10. Description of Metabolon QC Samples

Type	Description	Purpose
MTRX	Large pool of human plasma maintained by Metabolon that has been characterized extensively.	Assure that all aspects of the Metabolon process are operating within specifications.
CMTRX	Pool created by taking a small aliquot from every customer sample.	Assess the effect of a non-plasma matrix on the Metabolon process and distinguish biological variability from process variability.
PRCS	Aliquot of ultra-pure water	Process Blank used to assess the contribution to compound signals from the process.
SOLV	Aliquot of solvents used in extraction.	Solvent Blank used to segregate contamination sources in the extraction.

Table S11. Metabolon QC Standards

Type	Description	Purpose
RS	Recovery Standard	Assess variability and verify performance of extraction and instrumentation.
IS	Internal Standard	Assess variability and performance of instrument.

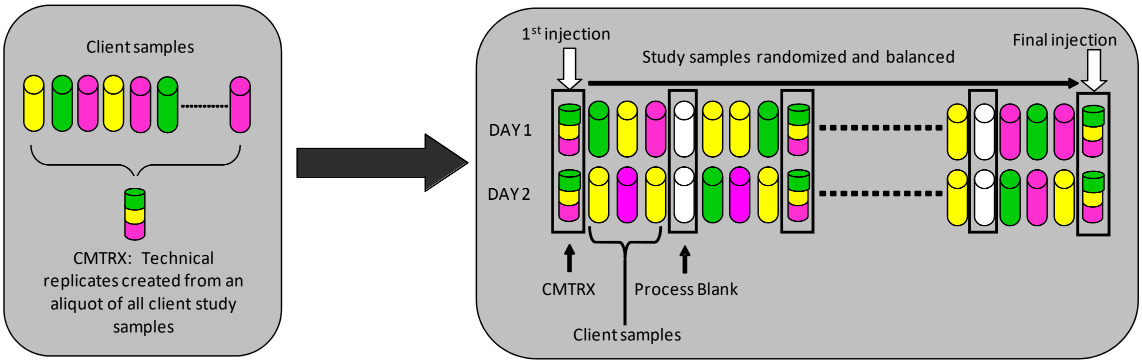

Figure S3. Preparation of client-specific technical replicates. A small aliquot of each client sample (colored cylinders) is pooled to create a CMTRX technical replicate sample (multi-colored cylinder), which is then injected periodically throughout the platform run. Variability among consistently detected biochemicals can be used to calculate an estimate of overall process and platform variability.

Ultrahigh Performance Liquid Chromatography-Tandem Mass Spectroscopy (UPLC-MS/MS): All methods utilized a Waters ACQUITY ultra-performance liquid chromatography (UPLC) and a Thermo Scientific Q-Exactive high resolution/accurate mass spectrometer interfaced with a heated electrospray ionization (HESI-II) source and Orbitrap mass analyzer operated at 35,000 mass resolution. The sample extract was dried then reconstituted in solvents compatible to each of the four methods. Each reconstitution solvent contained a series of standards at fixed concentrations to ensure injection and chromatographic consistency. One aliquot was analyzed using acidic positive ion conditions, chromatographically optimized for more hydrophilic compounds. In this method, the extract was gradient eluted from a C18 column (Waters UPLC BEH C18-2.1x100 mm, 1.7 µm) using water and methanol, containing 0.05% perfluoropentanoic acid (PFPA) and 0.1% formic acid (FA). Another aliquot was also analyzed using acidic positive ion conditions, however it was chromatographically optimized for more hydrophobic compounds. In this method, the extract was gradient eluted from the same afore mentioned C18 column using methanol, acetonitrile, water, 0.05% PFPA and 0.01% FA and was operated at an overall higher organic content. Another aliquot was analyzed using basic negative ion optimized conditions using a separate dedicated C18 column. The basic extracts were gradient eluted from the column using methanol and water, however with 6.5mM Ammonium Bicarbonate at pH 8. The fourth aliquot was analyzed via negative ionization following elution from a HILIC column (Waters UPLC BEH Amide 2.1x150 mm, 1.7 µm) using a gradient consisting of water and acetonitrile with 10mM Ammonium Formate, pH 10.8. The MS analysis alternated between MS and data-dependent MS^n scans using dynamic exclusion. The scan range varied slighted between methods but covered 70-1000 m/z. Raw data files are archived and extracted as described below.

Bioinformatics: The informatics system consisted of four major components, the Laboratory Information Management System (LIMS), the data extraction and peak-identification software, data processing tools for QC and compound identification, and a collection of information interpretation and visualization tools for use by data analysts. The hardware and software foundations for these informatics components were the LAN backbone, and a database server running Oracle 10.2.0.1 Enterprise Edition.

LIMS: The purpose of the Metabolon LIMS system was to enable fully auditable laboratory automation through a secure, easy to use, and highly specialized system. The scope of the Metabolon LIMS system encompasses sample accessioning, sample preparation and instrumental analysis and reporting and advanced data analysis. All of the subsequent software systems are grounded in the LIMS data structures. It has been modified to leverage and interface with the in-house information extraction and data visualization systems, as well as third party instrumentation and data analysis software.

Data Extraction and Compound Identification: Raw data was extracted, peak-identified and QC processed using Metabolon's hardware and software. These systems are built on a web-service platform utilizing Microsoft's .NET technologies, which run on high-performance application servers and fiber-channel storage arrays in clusters to provide active failover and load-balancing. Compounds were identified by comparison to library entries of purified standards or recurrent unknown entities. Metabolon maintains a library based on authenticated standards that contains the retention time/index (RI), mass to charge ratio (*m/z)*, and chromatographic data (including MS/MS spectral data) on all molecules present in the library. Furthermore, biochemical identifications are based on three criteria: retention index within a narrow RI window of the proposed identification, accurate mass match to the library +/- 10 ppm, and the MS/MS forward and reverse scores between the experimental data and authentic standards. The MS/MS scores are based on a comparison of the ions present in the experimental spectrum to the ions present in the library spectrum. While there may be similarities between these molecules based on one of these factors, the use of all three data points can be utilized to distinguish and differentiate biochemicals. More than 3300 commercially available purified standard compounds have been acquired and registered into LIMS for analysis on all platforms for determination of their analytical characteristics. Additional mass spectral entries have been created for structurally unnamed biochemicals, which have been identified by virtue of their recurrent nature (both chromatographic and mass spectral). These compounds have the potential to be identified by future acquisition of a matching purified standard or by classical structural analysis.

Curation: A variety of curation procedures were carried out to ensure that a high quality data set was made available for statistical analysis and data interpretation. The QC and curation processes were designed to ensure accurate and consistent identification of true chemical entities, and to remove those representing system artifacts, mis-assignments, and background noise. Metabolon data analysts use proprietary visualization and interpretation software to confirm the consistency of peak identification among the various samples. Library matches for each compound were checked for each sample and corrected if necessary.

Metabolite Quantification and Data Normalization: Peaks were quantified using area-under-the-curve. For studies spanning multiple days, a data normalization step was performed to correct variation resulting from instrument inter-day tuning differences. Essentially, each compound was corrected in run-day blocks by registering the medians to equal one (1.00) and normalizing each data point proportionately (termed the "block correction"; Figure 2). For studies that did not require more than one day of analysis, no normalization is necessary, other than for purposes of data visualization. In certain instances, biochemical data may have been normalized to an additional factor (e.g., cell counts, total protein as determined by Bradford assay, osmolality, etc.) to account for differences in metabolite levels due to differences in the amount of material present in each sample.

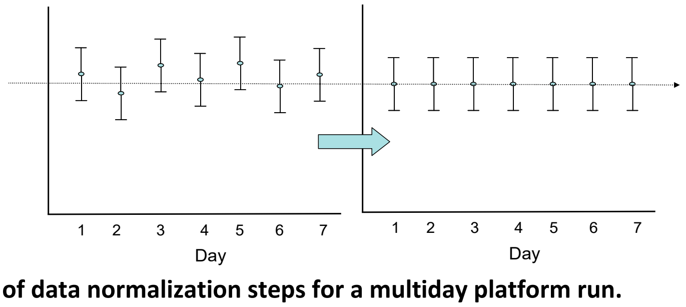

Figure S4. Visualization of data normalization steps for a multiday platform run.

Statistical Methods and Terminology

Statistical Calculations: For many studies, two types of statistical analysis are usually performed: (1) significance tests and (2) classification analysis. Standard statistical analyses are performed in ArrayStudio/Jupyter Notebook on log transformed data. For those analyses not standard in ArrayStudio/Jupyter Notebook, the programs R (http://cran.r-project.org/) or JMP are used. Below are examples of frequently employed significance tests and classification methods followed by a discussion of p- and q-value significance thresholds.

1. **One-way ANOVA**

   ANOVA stands for analysis of variance. For ANOVA, it is assumed that all populations have the same variances. One-way ANOVA is used to test whether at least two unknown means are all equal or whether at least one pair of means is different. For the case of two means, ANOVA gives the same result as a two-sided *t*-test with a pooled estimate of the variance.

   An ANOVA uses an F-test which has two parameters – the numerator degrees of freedom and the denominator degrees of freedom. The degrees of freedom in the numerator are equal to $g - 1$, where g is the number of groups. If n is the total number of observations ($n_1 + n_2$), then, the denominator degrees of freedom is equal to $n - g$. The F-statistic is the ratio of the between-groups variance to the within-groups variance, hence the higher the F-statistic the more evidence we have that the means are different.

   Often within ANOVA, one performs linear contrasts for specific comparisons of interest. For example, suppose we have three groups A, B, C, then examples of some contrasts are A vs. B, the average of A and B vs. C, etc. For single-degree of freedom contrasts, these give the same result as a two-sided *t*-test with the pooled estimate of the variance from the ANOVA and degrees of freedom $n - g$. Below, we show the three formulas for A vs. B from a three group design as shown above. The numerator is same in each case, but the denominator differs by the estimates of the variances, and the degrees of freedom are different for each (if the theoretical assumptions hold, then the contrast has the most power, as it has the largest degrees of freedom).

   Welch's two-sample *t*-test

   By $t = (\bar{x}_A - \bar{x}_B)/\sqrt{s_A^2/n_A + s_B^2/n_B}$, and the degrees of freedom is given by $\left(\frac{s_A^2}{n_A} + \frac{s_B^2}{n_B}\right)^2 / \left(\frac{\left(\frac{s_A^2}{n_A}\right)^2}{n_A - 1} + \frac{\left(\frac{s_B^2}{n_B}\right)^2}{n_B - 1}\right)$

   Two-sample *t*-test with pooled estimate of variance from A and B

   $$t = (\bar{x}_A - \bar{x}_B)/\sqrt{s_{AB}^2(1/n_A + /n_B)}$$

   where $s_{AB}^2 = \left((n_A - 1)s_A^2 + (n_B - 1)s_B^2\right)/(n_A + n_B - 2)$, where the degrees of freedom is $n_A + n_B - 2$.
   The contrast from the ANOVA,

   $$t = (\bar{x}_A - \bar{x}_B)/\sqrt{s^2(1/n_A + /n_B)}$$

   where $s^2 = \left((n_A - 1)s_A^2 + (n_B - 1)s_B^2 + (n_C - 1)s_C^2\right)/(n_A + n_B + n_C - 3)$, where the degrees of freedom is given by where the degrees of freedom is $n_A + n_B + n_C - 3$.

2. **Correlation**

   Correlation measures the strength and direction of a *linear* association between two variables. The statistical test for correlation tests whether the true correlation is zero or not.

The square of the correlation is the percentage of the total variation explained by a linear relationship between the two variables. Thus, with large sample sizes there may be a sample correlation of 0.1 that is statistically significant. This means we have high confidence that the true correlation is zero, however, only 100*(0.1*0.1)% = 1% of the variation of one variable is explained by a linear relationship with the other variable, so while there is an association, it has little predictive ability.

3. p-values

For statistical significance testing, p-values are given. The lower the p-value, the more evidence we have that the null hypothesis (typically that two population means are equal) is not true. If "statistical significance" is declared for p-values less than 0.05, then 5% of the time we incorrectly conclude the means are different, when actually they are the same.

The p-value is the probability that the test statistic is at least as extreme as observed in this experiment given that the null hypothesis is true. Hence, the more extreme the statistic, the lower the p-value and the more evidence the data gives against the null hypothesis.

4. q-values

The level of 0.05 is the false positive rate when there is one test. However, for a large number of tests we need to account for false positives. There are different methods to correct for multiple testing. The oldest methods are family-wise error rate adjustments (Bonferroni, Tukey, etc.), but these tend to be extremely conservative for a very large number of tests. With gene arrays, using the False Discovery Rate (FDR) is more common. The family-wise error rate adjustments give one a high degree of confidence that there are zero false discoveries. However, with FDR methods, one can allow for a small number of false discoveries. The FDR for a given set of compounds can be estimated using the q-value (see Storey J and Tibshirani R. (2003) Statistical significance for genomewide studies. Proc. Natl. Acad. Sci. USA 100: 9440-9445; PMID: 12883005).

In order to interpret the q-value, the data must first be sorted by the p-value then choose the cutoff for significance (typically p<0.05). The q-value gives the false discovery rate for the selected list (i.e., an estimate of the proportion of false discoveries for the list of compounds whose p-value is below the cutoff for significance). For Table 3 below, if the whole list is declared significant, then the false discovery rate is approximately 10%. If everything from Compound 079 and above is declared significant, then the false discovery rate is approximately 2.5%.

Compound	*p*-value	*q*-value
Compound 103	0.0002	0.0122
Compound 212	0.0004	0.0122
Compound 076	0.0004	0.0122
Compound 002	0.0005	0.0122
Compound 168	0.0006	0.0122
Compound 079	0.0016	0.0258
Compound 113	0.0052	0.0631
Compound 050	0.0053	0.0631
Compound 098	0.0061	0.0647
Compound 267	0.0098	0.0939

Table S12. Example of q-value interpretation
